# Enzymatic Degradation of the Most Common Aliphatic Bio-Polyesters and Evaluation of the Mechanisms Involved: An Extended Study

**DOI:** 10.3390/polym14091850

**Published:** 2022-04-30

**Authors:** Antonella Rosato, Angela Romano, Grazia Totaro, Annamaria Celli, Fabio Fava, Giulio Zanaroli, Laura Sisti

**Affiliations:** Department of Civil, Chemical, Environmental and Materials Engineering, University of Bologna, Via Terracini 28, 40131 Bologna, Italy; antonellarosato@hotmail.it (A.R.); angela.romano6@unibo.it (A.R.); grazia.totaro@unibo.it (G.T.); annamaria.celli@unibo.it (A.C.); fabio.fava@unibo.it (F.F.); laura.sisti@unibo.it (L.S.)

**Keywords:** aliphatic bio-polyesters, enzymatic degradation, lipase, cutinase, proteinase K, surface erosion, endo/exo-type action mode

## Abstract

Commercial hydrolytic enzymes belonging to different subclasses (several lipases, proteinase k, cutinase) were investigated for their ability to degrade different aliphatic polyesters, i.e., poly(butylene succinate) (PBS), poly(butylene succinate-*co*-adipate) (PBSA), two poly(caprolactone), having two different molecular weights, poly(lactic acid) (PLA) and poly(propylene carbonate) (PPC). The enzyme screening was first carried out by investigating the capacity of fully degrading the target polymers in 24 h, then weight loss measurements of selected polyesters and target enzymes were performed. Solid residues after enzyme degradation were characterized by proton nuclear magnetic resonance (^1^H NMR), gel permeation chromatography (GPC), infrared spectroscopy (FT-IR), differential scanning calorimetry (DSC) and thermogravimetry (TGA). Liquid fractions were studied via GPC, ^1^H NMR and high-performance liquid chromatography (HPLC). PCL and PBSA were found to be the most biodegradable polyesters, under the conditions used in this study. PBS was fully degraded only by cutinase, whereas none of the tested enzymes were able to completely degrade PLA and PPC, in the conditions assessed here. Cutinase exhibited the highest hydrolytic activity on PBSA, while lipase from *Candida* sp. (CALB) on low molecular weight PCL. Chemical analyses on residual solids showed that the enzymatic degradation occurred homogeneously from the surface through an erosion mechanism and did not significantly affect the macromolecular structure and thermal stability. Cleaving action mode for each enzyme (endo- and/or exo-type) on the different polyesters were also proposed based on the evaluation of the degradation products in the liquid fraction.

## 1. Introduction

The continuous growth of the plastic production and consumption over the world has become a critical problem. Most common plastic materials, such as poly(ethylene) (PE), poly(styrene) (PS), poly(propylene) (PP), and poly(vinyl chloride) (PVC) are totally derived from fossil raw material and are not biodegradable [1]. The accumulation of such materials, at their end-of-life, in the environment, as well as their incineration producing CO_2_ and dioxins, negatively affect the ecosystems, resulting in an increase of both environmental pollution and global warming [2,3].

In the last decades, therefore, biodegradable polymers have received growing attention for application in several fields including agriculture, packaging and medicine, as one of the most effective and eco-friendly approaches to overcome the problem of plastic waste [4]. Aliphatic polyesters represent the majority of the biodegradable plastics given their high propensity to undergo enzymatic hydrolysis and degradation by several microorganisms [5]. Poly(butylene succinate) (PBS), poly(butylene succinate-*co*-adipate) (PBSA), poly(caprolactone) (PCL), poly(lactic acid) (PLA), poly(propylene carbonate) (PPC) and poly(hydroxyalkanoates) (PHAs) are among the most promising biodegradable commercial polyesters [6,7,8]. The chemical composition and structure, along with the physical properties, such as crystallinity, melting temperature (T_m_) and glass transition temperature (T_g_), and the surface properties, such as hydrophobicity and roughness, can significantly affect the degradation rate of these polymers [9,10]. In particular, the degradability of the polymers tends to increase as their T_m_ and T_g_ decrease [7]. Furthermore, the copolymerization usually promotes the biodegradability of polyesters, probably reducing their crystallinity [11]. In fact, studies report that polymers with lower crystallinity present greater hydrolysis rate, since the amorphous domains are more vulnerable to biodegradation [12].

Several hydrolytic enzymes, i.e., lipases, esterases, cutinases and proteases, have been investigated for their capability to catalyze the hydrolysis of different aliphatic polyesters [4,13,14,15,16]. Such enzymes usually show differences in substrate specificity and/or interfacial activation. The conformation and the surrounding of their active site diverge remarkably among the different hydrolases, although they preserve the typical catalytic triad, commonly consisting of serine, histidine and aspartate [17]. Two different types of enzymatic polymer degradation mechanisms are possible. The polymers can undergo either “bulk degradation”, consisting of the hydrolysis in the center of the bulk material that is accompanied by a substantial reduction of their molecular weight, or “superficial erosion”, consisting of the loss of the polymeric materials only from the surface [10]. The last type mainly involves hydrophobic and highly crystalline homopolymers that become smaller (due to loss of material from surface), while maintaining their mechanical and structural integrity [18]. Moreover, the enzymes can be able to hydrolyze the polymer mainly into monomers or low molecular water-soluble oligomers, thus acting by exo-type chain-end scission [19]. On the other hand, they can hydrolyze ester bonds randomly along the main chain of the polymer (endo-type degradation), producing oligomers as the main degradation products [20,21].

Lipases and esterases are mainly differentiated based on their substrate preference; the lipases are more active against water-insoluble long chain triacylglycerol, whereas the esterases preferentially hydrolyze water-soluble short acyl chain esters [22]. However, several studies reported many lipases and esterases as effective biocatalysts in the degradation of different aliphatic polyesters. In particular, bacterially extracted lipases from *Pseudomonas* [23,24,25,26], *Lactobacillus* [27] and *Alcaligenes* [4] genera, fungal-extracted lipases from *Candida* [28,29,30,31], *Aspergillus* [32], *Mucor* [15] and *Rhizopus* [4] genera, as well as hog liver esterase [7] resulted among the most effective enzymes in aliphatic polyesters degradation. Many of them are able to fully hydrolyze PBS, PBSA and PCL films within a few days or hours [4,6,33], typically following a surface degradation mechanism [34].

Cutinase is a serine hydrolase presenting both lipase and esterase characteristics; this enzyme generally hydrolyzes the cutin, an insoluble polymeric structural component of plant cuticles, consisting of hydroxy fatty acids (usually C16 or C18) that contains up to three hydroxyl groups, and exhibits low interfacial activation contrary to lipase [35]. In the last few years, cutinases from several fungal sources, such as *Alternaria brassicicola*, *Humicola insolens, Thermobifida cellulosilyca* and *Fusarium solani*, have been investigated in the degradation of different synthetic aliphatic polyesters [13,36,37]. In particular, Shi et al. [20] reported a surface erosion mechanism and an endo-type action mode for the PBS degradation by a recombinant cutinase, reaching 66.1% and nearly 100% PBS weight loss after 6 and 26 h degradation, respectively.

Proteases are hydrolytic enzymes able to break the peptide bonds within proteins, and can be distinguished into acid, neutral and alkaline [38]. Several serine proteases, such as α-chymotrypsin, trypsin, elastase, proteinase K, and subtilisin, were investigated in the degradation of different aliphatic polyesters [7], resulting in being more efficient in the degradation of poly(L-lactic acid) [5]. Proteinase K and many proteases from *Bacillus* sp. showed considerable polylactide-degrading activity [14,39]. In particular, PLA depolymerizing proteases have been shown to have a different enantioselectivity compared to lipase-type hydrolases toward poly(L-lactic acid) (PLLA) and poly(D-lactic acid)(PDLA), being able to hydrolyze only PLLA, whereas the lipase-type (including cutinase-like enzyme) preferentially hydrolyze PDLA [40].

**Figure 1:**
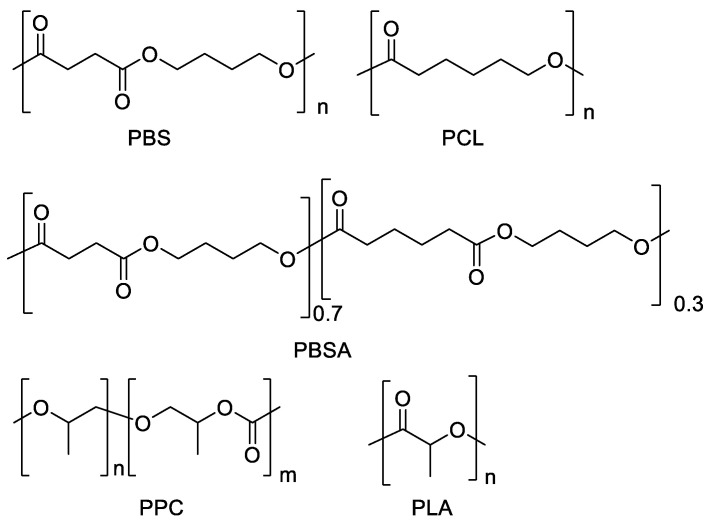
Chemical structures of PBS, PCL, PBSA, PPC and PLA.

As pointed out, among the most promising biodegradable commercial polyesters, which can be employed in diversified fields, PBS, PBSA, PCL, PLA and PPC can be found (Figure 1). In more detail, PBS is a semicrystalline polyester derived from 1,4-butanediol and succinic acid, both obtainable from sugar fermentation. It shows interesting properties, such as good tensile and impact strength, modest rigidity and hardness, but it has low melt strength and viscosity, and low gas barrier properties [41,42]. PBSA is a semicrystalline copolymer of PBS, with adipic acid as co-monomer, which is also potentially bio-based. With respect to PBS it has higher biodegradability due to its lower crystallinity and higher polymer chains flexibility. A molar feed ratio between succinate and adipate equal to 80/20 is indicated to be the best balance between the biodegradation rate and thermomechanical properties [43]. PCL is a semicrystalline polymer as well, prepared from the potentially bio-based cyclic ester ε-caprolactone or 6-hydroxyhexanoic acid. It is miscible with many other polymers, such as poly(vinyl chloride), poly(styrene acrylonitrile), poly(acrylonitrile butadiene styrene), etc., and it is also physically compatible with polyethylene, polypropylene, natural rubber, poly(vinyl acetate), and so on [34,44,45]. PLA can be prepared from L-lactic or D-lactic isomers, or a racemic mixture of both, obtaining poly-L-lactic acid (PLLA), poly-D-lactic acid (PDLA), or poly-D,L-lactic acid (PDLLA), respectively. The stereochemistry affects the material properties: PLLA is semicrystalline, while PDLLA is an amorphous polymer [46]. PPC is a regular alternating amorphous copolymer prepared from CO_2_ and the most reactive cyclic ether propylene oxide. PPC has high oxygen barrier properties but poor mechanical properties and thermal stability [47].

Although aliphatic polyesters cannot completely replace the conventional plastics due to their limited physical and mechanical properties [48], deeper elucidation on the degradative capability and the mechanism of hydrolytic biodegradation can be significantly useful for the management of biodegradable plastic wastes, the bioremediation of plastic-polluted environments, as well as the design of innovative biodegradable plastic materials. In this study, several commercial hydrolytic enzymes (11 lipases, 1 cutinase and 1 proteinase) were investigated for their ability to degrade PBS, PBSA, two PCL with different molecular weight, PLA and PPC. Many researches have been published on the enzymatic degradation of bio-polyesters in the last years. However, the present manuscript represents an extended study allowing to rapidly identify, among various commercial hydrolytic enzymes, the most effective in the hydrolysis of different types of polyesters, as well as to quickly compare their enzymatic degradation mechanisms. Indeed, a clear comparison of already reported results is quite difficult because of the great variability in the conditions tested. With the current extended study, such issue is overcome. A preliminary screening was here assessed by evaluating the degradation activity in 24 h; then, for the most biodegradable polymers, weight loss measurements were performed on target enzymes. Analyses on the solid residues of films at specific degradation times (Gel Permeation Chromatography GPC, proton nuclear magnetic resonance ^1^H NMR, infrared spectroscopy FT-IR, calorimetric analysis DSC, thermogravimetry TGA), as well as on the liquid media (high-performance liquid chromatography HPLC) for all degradation times were carried out. The last liquid fraction was further extracted in the organic phase and analyzed (GPC, ^1^H NMR). Based on the analyses conducted the enzymatic degradation mechanisms of PBS, PBSA and PCL polyesters were proposed.

## 2. Materials and Methods

### 2.1. Polyester Samples and Their Characterization Measurements

Six polyesters, i.e., poly(butylene succinate) (PBS, trade name FZ91PM, melt flow rate MFR (190 °C, 2.16 kg) = 5 g/10 min), poly(butylene succinate-*co*-adipate) (PBSA, trade name FD92PM, MFR (190 °C, 2.16 kg) = 4 g/10 min), two poly(caprolactone) (PCL6500D, trade name Capa™ 6500D, melt flow index MFI = 6.31 g/10 min, and PCL6800D, trade name Capa™ 6800D, MFI = 2.44 g/10 min), poly(lactic acid) (PLA, trade name Luminy^®^ LX175, MFI (190 °C/2.16 kg) = 3 g/10 min) and poly(propylene carbonate) (PPC, trade name QPAC^®^ 40). PBS and PBSA were purchased by PTT MCC Biochem, PCL by Ingevity, PLA by Corbion and PPC by Empower Materials.

From technical data sheets, PLA contains 96% of L-isomer, PPC contains PC monomer (3.6%).

The polymers (Figure 1) were characterized from a molecular, chemical and thermal point of view by ^1^H NMR, GPC, FT-IR, DSC and TGA. PPC was further analyzed by ^13^C NMR and Heteronuclear Single Quantum Coherence (HSQC-2D).

The ^1^H NMR spectra were acquired using a Varian Mercury 400 spectrometer, the chemical shifts were reported in ppm downfield from tetramethylsilane (TMS); the solvent used was CDCl_3_. The ^13^C NMR spectrum was acquired using a Varian Inova 600 spectrometer, with a ^13^C frequency of 125 MHz. HSQC (Heteronuclear Single Quantum Coherence) was also recorded by the same apparatus. PBS: ^1^H NMR (CDCl_3_, δ ppm), 400 MHz: 1.67–1.74 (4H, m, C^c^H_2_), 2.59–2.62 (4H, s, C^a^H_2_), 3.40–3.43 (4H, t, CH_2_), 3.65–3.70 (6H, m, CH_2_), 4.08–4.15 (4H, t, C^b^H_2_). PBSA: ^1^H NMR (CDCl_3_, δ ppm), 400 MHz: 1.65–1.67 (4H, m, C^e^H_2_), 1.70–1.71 (4H, m, C^b^H_2_), 2.31–2.33 (4H, t, C^d^H_2_), 2.62 (4H, s, C^c^H_2_), 3.41–3.44 (4H, t, CH_2_), 3.66–3.69 (2H, t, CH_2_OH), 4.09–4.12 (4H, t, C^a^H_2_). PCL6500D and PCL6800D: ^1^H NMR (CDCl_3_, δ ppm), 400 MHz: 1.35–1.42 (2H, m, C^c^H_2_), 1.61–1.69 (4H, m, C^b,d^H_2_), 2.29–2.33 (2H, t, C^e^H_2_), 3.63–3.68 (2H, t, CH_2_OH), 4.04–4.08 (2H, t, C^a^H_2_). PLA: ^1^H NMR (CDCl_3_, δ ppm), 400 MHz: 1.57–1.59 (3H, d, C^b^H_3_), 4.33–4.38 (1H, q, CHOH), 5.14–5.19 (1H, q, C^a^H). PPC: ^1^H NMR (CDCl_3_, δ ppm), 600 MHz: 1.26–1.27 (3H, d, C^f^H_3_), 1.33–1.34 (3H, d, C^c^H_3_), 3.47–3.57 (2H, m, C^e^H_2_), 3.70–3.81 (1H, m, C^d^H), 4.02–4.29 (2H, m, C^a^H_2_), 4.85–5.00 (1H, m, C^b^H); ^13^C NMR (CDCl_3_, δ ppm), 600 MHz: 16.09–16.25 (C^c^H_3_), 68.76–69.58 (C^b^H_2_), 71.82–72.48 (C^a^H), 153.62–154.66 (C=O). The carbon labels are referred to the chemical structure reported in Figures S1a–S3a, S4 and S5a,b, ESI.

Number average molecular weight (M_n_), weight average molecular weight (M_w_) and polydispersity index (PD) were measured by gel permeation chromatography (GPC) performed in CHCl_3_ at ambient temperature, using a Knauer Azura apparatus with a PL gel 5 µm Minimixed-C column and a refractive index detector. Polystyrene standards were employed for preparing a universal calibration curve. The lower limit of the operating range of the column was 500 g/mol.

FT-IR analysis was performed over the wavenumber range of 650–4000 cm^−1^ using a Perkin Elmer Spectrum One spectrometer equipped with a Universal ATR Sampling Accessory. Each spectrum was obtained from 32 scans.

The calorimetric analysis (DSC) was carried out using a Perkin-Elmer DSC6. Measurements were conducted under nitrogen flow. The first step was conducted to delete the thermal history of samples (around 10 mg) and it was performed by heating the sample from ambient temperature to the melting (at 20 °C min^−1^), kept at high temperature for 2 min and then cooled down to maximum −70 °C (at 10 °C min^−1^). Then, the 2nd scan was carried out and the samples were heated up to the melting (at 10 °C min^−1^). The temperature of crystallization (T_c_) and the enthalpy of crystallization (∆H_c_) were measured during the cooling scan, while the glass transition temperature (T_g_), the cold crystallization (T_cc_), the enthalpy of cold crystallization (ΔH_cc_), the melting temperature (T_m_), the enthalpy of fusion (∆H_m_) were measured during the 2nd heating scan. T_g_ was taken as the midpoint of the heat capacity increment associated with the glass-to-rubber transition. The degree of crystallinity was calculated as

(1) $$\chi_{c}\left(\%\right)=\frac{{\Delta H}_{m}}{{\Delta H}_{m}^{0}}\times 100$$

where ΔH_m_ is the experimental melting enthalpy of the sample and ΔH^0^_m_ is the melting enthalpy of the pure crystalline polymers, i.e., 200 J/g for PBS [49], 142.0 J/g for PBSA [50,51] and 139.5 J/g for PCL [52].

Thermogravimetric analysis (TGA) was carried out under nitrogen flow (40 mL min^−1^) using a Perkin Elmer TGA4000 apparatus, in the range of 50–900 °C with a heating rate of 10 °C min^−1^. The onset degradation temperature (T_onset_) was measured from the intersections of the tangents of the initial points and the inflection points.

### 2.2. Enzymes and Degradability Tests

Different commercially available hydrolytic enzymes (11 lipases, 1 cutinase, and 1 protease) were purchased for this study and are listed in Table 1. The enzymes were used in the degradability tests without further purification.

The capability of the selected enzymes to degrade the different polyesters (i.e., PBS, PBSA, PCL6500D, PCL6800D, PLA and PPC) was preliminary investigated by evaluating the enzyme capacity of fully degrading the target polymers in 24 h. To this purpose rectangular polymers films (1 cm × 2.5 cm; 0.15 mm thickness), obtained by molding at 180 °C for PLA, 150 °C for PPC, 120 °C for PCL and 170 °C for PBSA, were incubated in sodium phosphate buffer 0.1 M (5 mL) in the presence of each enzyme at the concentration (U/mL) reported in Table 1. The enzymatic solutions were prepared according to the unit amount per mg of solid or mL of liquid enzyme product indicated by the suppliers. The incubation was performed in 10 mL screw-cap glass vials in a thermostatic bath under gentle mixing and under the optimal reaction conditions of each enzyme (Table 1). Negative controls (enzyme-free) were also set up by incubating each polymer film type in buffer solution under the same conditions. Polymer films were periodically monitored (i.e., after 0.3, 1, 2, 4, 6 and 24 h incubation) in order to check their complete degradation.

Degradation processes were further investigated via film weight loss test, which was performed only in the presence of the most active enzymes, i.e., those that were able to completely degrade the polymers in the preliminary assay. Table 1 shows the polyesters, the conditions, in terms of pH and temperature, the enzymes and their concentrations used in weight loss test. In particular, the enzyme concentration was reduced to 10 U/mL for all commercial enzymes, based on the high and rapid degradation observed in the preliminary assay. Lipases from *Pseudomonas fluorescens*, *Rhizopus oryzae*, *Candida* sp. (CALB), *Alcaligenes* sp. (QLM) and cutinase were tested on rectangular polymeric films (9 cm^2^ surface, 0.15 mm thickness) that were incubated in 18 mL of sodium phosphate buffer (0.1 M); tests were conducted in duplicate for each enzyme-polymer combination. The polymer surface area and the buffer volume were selected in order to maintain the same polymer surface/enzyme solution ratio used in the preliminary screening. However, a sufficient number of units was not available for lipase from *Pseudomonas* sp. and lipase B from *Candida antarctica*, which were tested on films having 1 cm^2^ of surface in 2 mL of enzyme solution (thus keeping the same polymer surface/enzyme solution ratio); tests with these enzymes were also performed in single replicates. In all cases, optimal reaction conditions of each enzyme were used (Table 1). The tests were carried out in 20 mL or 5 mL screw-cap glass vials for larger and smaller films, respectively. Enzyme-free controls were also set up by incubating polyester (PBS, PBSA, PCL6500D and PCL6800D) films in buffer solution without enzymes under the same conditions. Polymer films were dried overnight under vacuum and weighed before enzyme incubation. At different time intervals, sacrificial polymeric films were washed with deionized water, dried overnight under vacuum, at ambient temperature, and then weighted. The weight loss was calculated by subtracting the dry weight remaining at a specific sampling time to the initial one. Linear regression analyses were calculated by plotting the amount (mg) of each degraded polymer over time; the resulting value was then divided by the polymer surface area in order to determine the enzymatic degradation rate (mg_degraded polymer_/h/cm^2^).

### 2.3. Evaluation of Polyester Enzymatic Degradation Mechanisms

In order to investigate the possible enzymatic degradation mechanisms of the polyesters, several chemical analyses were performed on the polymer solid residues of the weight loss tests. In particular, some selected solid residues of PBS, PBSA and PCL polyesters, treated with different enzymes, were analyzed by FT-IR, DSC, TGA, ^1^H NMR and GPC. The measurements were conducted as described in Section 2.1. However, some data are not available because the amount of some samples was insufficient for the spectroscopic analysis.

The liquid fraction of the weight loss tests was also subjected to a number of analyses in order to identify the release of oligomers and monomers during the polymer degradation. In particular, the last sample of incubation was extracted in CH_2_Cl_2_ (3 × 15 mL), dried with MgSO_4_, evaporated at reduced pressure and further analyzed by GPC and ^1^H NMR, in order to evaluate the presence of oligomers and some monomers, i.e., those soluble in CH_2_Cl_2_. In particular, 6-hydroxy hexanoic acid (HA) and 1,4-butanediol (BD) are soluble both in water and organic phase; conversely, succinic acid (SA) and adipic acid (AA) remained in the aqueous phase, since they are not soluble in CH_2_Cl_2_. In some cases, the amount of sample was not enough to proceed and, therefore, the extraction was not carried out. The monomers released in the liquid fraction of the weight loss tests were also identified and measured at each sampling time via high-performance liquid chromatography (1260 Infinity, Agilent Technologies, Santa Clara, CA, USA) equipped with a Refractive Index Detector (HPLC-RID), an automatic sampler and a Hi-Plex H column (Agilent Technologies). Analyses were carried out employing an isothermal and isocratic method under the following conditions: detector temperature = 40 °C; column temperature = 65 °C; mobile phase = deionized water + 0.005% (*w*/*v*) sulfuric acid; flow rate = 0.6 mL/min. SA, AA, BD and HA were identified and constantly calibrated (from 0.05 g/L to 1 g/L) using commercial standard.

## 3. Results and Discussion

### 3.1. Polyester Characterizations

Molecular and thermal properties of polyesters are reported in Table 2. All polyesters presented high M_w_ around 200,000 g/mol and polydispersity values consistent with those expected from commercial samples. PCL6500D and PCL6800D resulted to have 153,000 and 216,000 g/mol as M_w_, respectively. From DSC analysis, PLA and PPC are amorphous, with T_g_ of 59 and 17 °C, respectively. PLA, during the second heating scan, just above the T_g_, can crystallize at 116 °C (T_cc_) and then melts at 152 °C. Such cold crystallization is due to enhanced primary nucleation occurring above the glass transition temperature when polymer chains have enough energy to move and achieve an ordered state [53] (Sisti et al. 2016). On the other hand, PBS, PBSA and PCL resulted semicrystalline, with melting at 116, 87 and 58 °C, respectively. Curves are showed in ESI, Figure S6.

**Figure 2:**
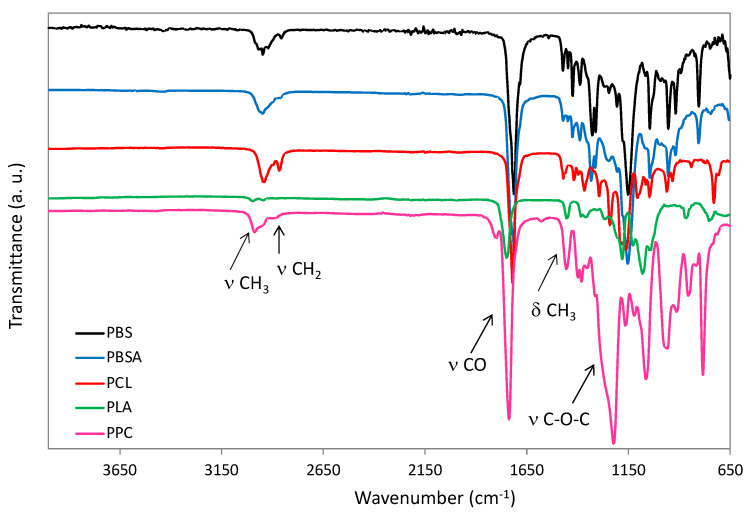
ATR FT-IR curves of PBS, PBSA, PCL, PLA and PPC.

All samples showed high thermal stability, with T_onset_ values, determined by TGA, in the range 240–390 °C (ESI, Figure S7a,b). IR curves (Figure 2) are consistent with literature [54,55,56]. ^1^H NMR analysis confirms the chemical structures (ESI, Figures S1a–S3a, S4 and S5a). Concerning PBSA, it resulted by ^1^H NMR analysis being a copolymer with the following composition: (PBS)_0.74_–(PBA)_0.26_. The ratio BS/BA has been evaluated by the comparison of the peak around 2.6 ppm and 2.3 ppm (ESI, Figure S2a). For an exact assignment of the peaks, PPC was further analyzed by ^13^C NMR and Heteronuclear Single Quantum Coherence (HSQC-2D) (ESI, Figure S5b,c). Additional comments about PPC, the details and spectra of all samples, are reported in ESI.

### 3.2. Degradation of Polyesters by Hydrolytic Enzymes

#### 3.2.1. Enzymes General Screening

Several commercially available hydrolytic enzymes belonging to different subclasses (i.e., 11 lipases, 1 cutinase and 1 proteinase) were tested for their ability to hydrolyze the esters bonds occurring in PBS, PBSA, two PCL with different M_w_ (PCL6500D and PCL6800D), PLA and PPC polyesters.

The enzymatic degradation was preliminary investigated by evaluating the capacity of fully degrading the target polymers in 24 h, which allowed to obtain a rapid overview of the enzymes potentially more active against the different polyesters. The main results of the assay are summarized in Table 3.

Particularly, a fast degradation of PBS film was observed only in the presence of cutinase after 2–4 h. Compared to PBS homopolymer, PBSA copolymer resulted being more enzymatically degradable. In particular, lipases QLM and cutinase completely hydrolyzed PBSA film after 1–2 h of incubation, lipase from *Pseudomonas* sp. after 4–6 h, and lipase from *Candida* sp. (CALB) within 24 h. No degradation of PBSA film occurred in the presence of lipase from hog pancreas lipase from *Candida rugosa*, lipase from *Candida cylindracea* and proteinase K. On the other hand, lipase B from *Candida antarctica* and lipase from *Pseudomonas fluorescens* were able to entirely degrade PBSA polyester, although in more than 24 h (Table 3). Concerning PCL, many enzymes were able to rapidly and completely degrade both different M_w_ PCL samples (Table 3). In particular, PCL6500D film was completely degraded by lipase CALB (after 0.3–1 h incubation), followed by lipase B from *Candida antarctica,* lipase QLM and cutinase (after 1–2 h), lipase from *Pseudomonas* sp. (after 2–4 h), and lipases from *Pseudomonas fluorescens* and *Rhizopus oryzae* (after 6–24 h). Similarly, PCL6800D was completely hydrolyzed by lipase CALB after 2–4 h incubation, followed by cutinase (after 4–6 h), and lipase B from *Candida antarctica* and lipase QLM (after 6–24 h). Lipases from *Pseudomonas fluorescens*, *Rhizopus oryzae* and *Pseudomonas* sp. were not able to degrade the high molecular weight PCL. These results indicate that PCL having higher M_w_ (PCL6800D) was less degradable by the tested enzymes compared to the one having lower M_w_ (PCL6500D), since the latter was fully hydrolyzed both in a shorter time and by a greater number of enzymes. This could be explained by more limited movements of the polymer chains occurring when the molecular weight increased from 153,000 (PCL6500D) to 216,000 g/mol (PCL6800D). The molecular weight effect on the enzymatic degradation of PCL polyester was also investigated by Li et al. [57], who observed a lower degradation rate for polyesters having higher M_w_.

Regarding PLA, none of the tested enzymes was able to completely degrade the polymer during the entire period of incubation, under the conditions used in this study (Table 3). Williams [58] revealed for the first time the degradation of PLA by the proteinase K from *Tritirachium album*. Further studies showed that enzymes belonging to the class of proteases could degrade PLLA, whereas enzymes belonging to lipases class preferentially degrade PDLA [14,40]. Even though the PLA polyester tested in this study consisted of 96% enantiomer “L”, no weight loss of the polymer film was observed after incubation with proteinase K. It is well known that proteases and lipases both belong to serine hydrolases, presenting the same catalytic triad (mainly serine, histidine and aspartate) in their active site. However, the topology of the catalytic triad in serine proteases is the mirror image of that in α/β hydrolase fold lipase, probably explaining their different substrate preference (PLLA vs PDLA, respectively) [14,59].

Concerning PPC polyester films no complete degradation was observed in the presence of all the tested enzymes (Table 3). In the same way, a study conducted by Hwang et al. [24] showed that lipases from *Pseudomonas* spp. and *Rhizopus arrhizus* exhibited very poor hydrolytic activity against PPC film (0.6 and 2.4% of degradation after 3 days incubation, respectively), compared to that against poly(propylene carbonate-*co*-ε-caprolactone) (65.3 and 10.0%, respectively) and PCL (78.2 and 13.2%, respectively). The negligible degradation activity observed on this polymer could be explained by the physical properties of PPC (such as high T_g_) and/or by the methyl substituents in the polyester backbone that inhibit the access of the PPC substrate to the enzymes active site [24].

In summary, this general screening revealed that PBSA and PCL were the most enzymatically degradable polyesters. In particular, PCL6500D and PCL6800D films were completely degraded by seven enzymes (six lipases and cutinase) and four enzymes (three lipase and cutinase), respectively, within an incubation time ranging from 0.3 up to 24 h. Five lipases and cutinase completely degraded also PBSA films, although during a generally longer incubation time than PCL polyesters. Conversely, PBS was completely degraded only by cutinase (within 4 h), and none of the tested enzymes was able to fully hydrolyze both PLA and PPC.

#### 3.2.2. Weight Loss Tests: Specific Polymers and Target Enzymes

**Figure 3:**
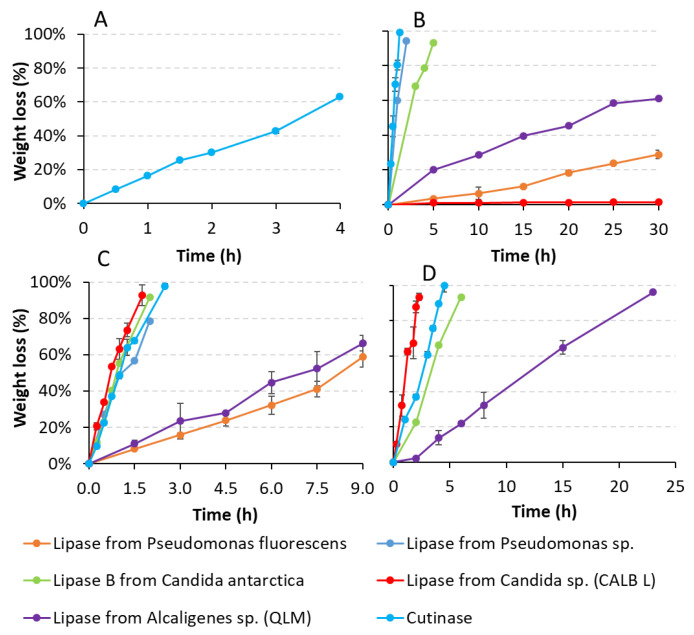
Weight loss (%) of PBS (**A**), PBSA (**B**), PCL6500D (**C**) and PCL6800D (**D**) films over time (hours) in the presence of the different enzymes.

In order to obtain more quantitative information, the degradation processes of PBS, PBSA and both PCL were further investigated via film weight loss tests, only in the presence of the most active enzymes (10 U/mL), i.e., those able to completely degrade the polymers in the previous general assay (Table 3). In particular, film weight loss of the polymers was monitored over time (Figure 3) and the relative degradation rates were calculated for each enzyme (Table 4). The films weight loss in the negative controls (enzyme-free) was negligible for PBSA (approximately 0.5%) and PCL6500D (approximately 0.2%), and completely absent for PBS and PCL6800D after 30 h of incubation in phosphate buffer (data not shown). Conversely, these polymers were rapidly and extensively hydrolyzed when incubated with many enzymes (Figure 3, Table 4). In particular, PBS film showed more than 60% weight loss in 4 h when exposed to cutinase (Figure 3A), corresponding to an initial degradation rate of 0.35 mg/h/cm^2^ (Table 4). PBSA films were more rapidly degraded by cutinase, showing approximately 6.38 mg/h/cm^2^ of degradation rate, followed by lipase from *Pseudomonas* sp. and lipase B from *Candida antarctica* (Table 4). Remarkably, such specific degradation rates led to a weight loss higher than 90% after only 1.3, 2 and 5 h of incubation, respectively (Figure 3B). A much lower PBSA degradation activity was exhibited by lipases from *Alcaligenes* sp. (QLM) and *Pseudomonas fluorescens*, reaching after 30 h incubation approximately 61 and 30% of weight loss, respectively. Negligible PBSA degradation (approximately 1% weight loss) occurred in the presence of lipase from *Candida* sp. (CALB) after 30 h incubation (Figure 3B, Table 4).

These degradative tests showed that the active site of cutinase was the most accessible to PBS chains in comparison with the other enzymes tested in this study, since cutinase was the only one able to efficiently degrade PBS polyester. Ping et al. [60] assumed that the active site of most cutinases is not covered, thus differing from that of the lipases. Recently, many studies have described similar PBS hydrolysis results using enzymes belonging to the cutinase family. Bai et al. [12] and Hu et al. [36] reported a PBS weight loss of 76.3% (after 4 h degradation) and 100% (within 6 h), respectively, in the presence of recombinant cutinases from *Fusarium solani* cloned and overexpressed in *Pichia Pastoris*. Extensive PBS weight loss (up to 41 and 92%) was also detected by Gamerith et al. [1] during 96 h of incubation with native and glycosylation site knock out mutant cutinase from *Thermobifida cellulosilyca* expressed in *P. pastoris*, respectively. Similarly, Shi et al. [20] observed that degradation rates of PBS by *P. pastoris* expressed cutinase from *Fusarium* sp. was higher than those by lipase from *Candida antarctica* (CALB) at the same enzyme concentrations, corresponding to PBS weight loss after 6 h incubation of approximately 66% and 37%, respectively. Furthermore, our degradative tests showed that PBSA copolymer was more easily and rapidly degradable compared to the PBS homopolymer. This is not surprising, since the copolymerization usually promotes the biodegradability of a polymer by lowering its crystallinity and melting temperature [11]. In particular, the introduction in PBS polyester of the adipate moiety (up to 60%) typically increases the chain mobility and thus biodegradability of PBSA [61], making this copolymer one of the most interesting biodegradable aliphatic polyesters [6].

No weight loss of PCL6500D occurred with lipase from *Rhizopus oryzae* after 6 days incubation (data not shown), by decreasing its concentration from 100 U/mL (the one used in the 24 h screening) to 10 U/mL (the one used in the weight loss test). Conversely, the other enzymes were able to efficiently degrade both PCL polymers. In particular, PCL6500D films were rapidly degraded in the presence of lipase CALB, exhibiting a degradation rate of approximately 5.2 mg/h/cm^2^. A similar degradation rate was also revealed by cutinase (approximately 4.6 mg/h/cm^2^), followed by lipase B from *Candida antarctica* and lipase from *Pseudomonas* sp. (Table 4). These values corresponded to weight losses higher than 90% after an enzymatic incubation time ranging from 1.3 to 2.5 h. Lipases from *Alcaligenes* sp. (QLM) and *Pseudomonas fluorescens* proved to be much less active against PCL6500D polyester, leading after 9 h of incubation to approximately 66% and 59% weight losses, respectively (Figure 3C); lower degradation rates were thus exhibited by these last two enzymes on this polyester (Table 4). Similarly, PCL6800D was rapidly hydrolyzed by lipase CALB with a degradation rate of 4.78 mg/h/cm^2^, corresponding to approximately 90% weight loss after 2.3 h incubation (Figure 3D). Although with lower degradation rates (Table 4), this polymer was also completely degraded (100% weight loss) by cutinase within 6 h of incubation (Figure 3D). More than 90% of film weight loss was also observed with lipase B from *Candida antarctica* within 6 h, whereas lipase from *Alcaligenes* sp. (QLM) resulted the least active enzyme against PCL6800D, producing approximately 96% of its weight loss only after 23 h of incubation.

Previous studies reported that PCL polyester can be efficiently and rapidly degraded by different lipases and cutinases. Gan et al. [23] showed a complete degradation of PCL within 4 days of incubation in phosphate buffer containing *Pseudomonas* lipase. Similarly, lipases from *Chromobacterium viscosum*, *Rhizopus niveus*, and *Alcaligenes* sp. were able to fully hydrolyze PCL films during 4–17 days of incubation [4]. Recently, Liu et al. [35] revealed that the degradation of PCL film could be enhanced with a bifunctional *T. lanuginosus* lipase and *T. terrestris* cutinase, reaching more than 90% of PCL degradation after 6 h. According to the results obtained in this study, Shi et al. [62] observed a PCL film weight loss with CALB lipase 2.64 times higher than that obtained with cutinase.

The enzymatic degradability of the aliphatic polyesters in this study has mainly increased according to the decrease of their melting point (T_m_) and glass transition temperature (T_g_) (Table 2), as already reported by several previous studies [7,12]. A low T_g_ indirectly suggests the presence of a mobile amorphous phase, which could accelerate the biodegradation [12]. In fact, the enzymes mainly exhibited the highest degradation rates against both PCL polyesters, which are the ones with the lowest T_m_ (less than 50 °C) and T_g_ (−61 °C) among the polymers tested in this study. The only exception was observed for cutinase that proved to be more efficient against PBSA, indicating that this copolymer is a better substrate for cutinase compared to PCL polyester. PBSA, having a T_m_ and T_g_ (87 and −45 °C, respectively) slightly higher than those of PCL, which was also rapidly and extensively degraded by many other enzymes. Conversely, PBS was efficiently degraded only by cutinase. In fact, PBS has a greater T_m_ and T_g_ (116 and −31 °C, respectively) than those of PBSA, thus confirming our assumption. The weight loss test was not performed on PLA since none of the tested enzymes in the general screening was able to completely hydrolyze it, such amorphous polymer has the highest T_g_ (59 °C) among the tested polyesters. Marten et al. [63] revealed that high polymer degradation rates only occur when the difference between the temperature used in the enzymatic hydrolysis and T_m_ is less than 30 °C. Since the optimum temperature of each enzyme used in these experiments was 30 or 40 °C, such ΔT (less than 30 °C) was generally maintained in the case of PCL. This could also explain how the highest PCL degradation rates occurred in the presence of many enzymes during the weight loss tests; moreover, it should be noted that the degradation rates of the material tested follows the same order of their melting temperatures. On the other hand, previous studies reported that the ratio CH_2_/CO in a polyester could be another dominant factor in controlling the enzymatic hydrolysis, since a higher ratio corresponds to higher flexibility, with consequent higher degradation. Fields et al. [64] showed that the aliphatic polyesters with C6 to C10 chains were more rapidly hydrolyzed than C4. Later, Bai et al. [12] confirmed that a decrease in polymer degradation rates is probably associated with a decrease in CH_2_/CO ratio; they observed that polyesters prepared with more methylene groups in the backbone presented higher hydrolysis by cutinase. These studies agree with the degradation results obtained in our experiments, in which the degradation rates occurred in the order PCL > PBSA > PBS, thus decreasing with the decrease in CH_2_/CO ratio.

### 3.3. Enzymatic Degradation Mechanisms of PBS, PBSA and PCL Polymers

#### 3.3.1. Solid Residue Films Characterization

In order to investigate the possible enzymatic degradation mechanism, several chemical analyses (^1^H NMR, GPC, FT-IR, DSC and TGA) were performed on selected solid residues of polyesters after incubation with the different enzymes at different time.

As a matter of fact, the macromolecular structures analysed by ^1^H NMR did not highlight substantial differences; the spectra related to the polyester films after cutinase degradation, as representative for all the enzymes, are showed in ESI, Figures S1b–S3b and S3g. In the case of PBSA films the ratio, BA/BS is always maintained after the enzymatic treatment, highlighting no specific preference of the enzyme with respect to one moiety. The percentage of OH-end groups was also evaluated by ^1^H NMR analysis as a ratio between the normalized integrals of peak of CH_2_OH end groups and the average value of the normalized integrals related to the protons present within the polymer chain (CH_2_OCO). The values slightly increased and their amount (around 1/2 mol%) was consistent with the high M_w_ resulting from GPC.

The amount of OH terminal group determined by NMR for all the samples at different sampling times are listed in Table 5. By comparing the molecular weights (M_n_ and M_w_) of the degraded samples respect to the control, the general trend of most polymers seems to highlight an initial decrease, more or less significant according to the enzyme and the target polymer, and then the values remain similar. In more detail, the M_n_ values in most cases decreased by about 20% in the early stages of incubation but successively, no further significant changes were detected. In the case of cutinase and lipase from *Candida* sp. (CALB) degraded samples, the differences concerning such first initial decrease of M_n_ are smaller (around 15% in PBSA and PCL6500D and less than 5% with PBS and PCL6800D). Bikiaris et al. [65] observed a similar trend in poly(alkylene succinate)s degraded by *Rhizopus delemar* lipase. The chains in the amorphous regions are more flexible and accessible by water, and therefore ester bonds can be hydrolysed more easily. When the enzymes reach the crystalline regions, it is more difficult for water to diffuse, and thus only short segments are removed from the end-chains, and the molecular weight is not affected until films are totally degraded. In general, this indicates that the degradation occurred homogenously on the surface through an erosion mechanism, as already reported in the literature [12,36,66].

**Figure 4:**
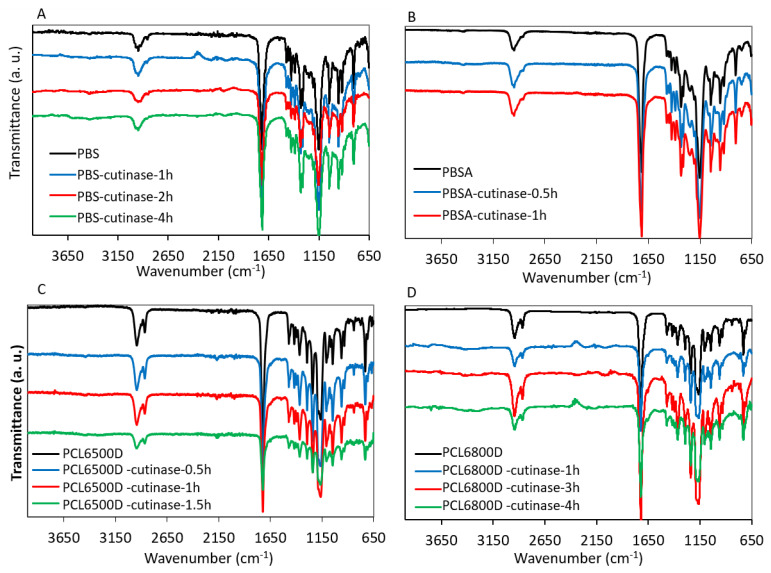
ATR FT-IR curves of PBS (**A**), PBSA (**B**), PCL 6500D (**C**) and PCL 6800D (**D**) residual solids after treatment with cutinase.

Infrared spectra of treated polyester films did not highlight significant differences compared to the pristine polymers. Figure 4 shows the infrared spectra of cutinase treated PBS (Figure 4A), PBSA (Figure 4B), PCL6500D (Figure 4C) and PCL6800D (Figure 4D) for example (the other curves are reported in ESI, Figure S8). The peaks at 1712 cm^−1^ and 1150 cm^−1^ in PBS and PBSA corresponding to the C=O and C-O-C stretching vibrations respectively are present before and after degradation [12]. Similarly, in the case of PCL6500D and PCL6800D, the stretching vibrations of C=O and C-O-C at 1721 cm^−1^ and 1163 cm^−1^ are always present. Shi et al. [62] associated such bands to the crystalline and amorphous regions of PCL respectively: since no significant differences or relative decrease are found in degraded curves with respect to non-degraded ones, it could be hypothesized that the degradation occurred indifferently in the amorphous and the crystalline regions.

The thermal properties of the degraded polyesters were analysed by DSC and TGA and were compared to the pristine polymers. The T_onset_ calculated from TGA (data not shown) did not show any significant differences in all polymers, indicating that degradation did not affect the thermal stability and that the polymer chains were not greatly damaged throughout the degradation process. The DSC results are listed in Table S1. T_m_ values of degraded films do not considerably change, while slight changes are present in T_c_ in PBSA and PCL6500D film samples treated with cutinase. Moreover, T_g_ of PBSA slightly decreases in the early stages of incubation but successively, no further significant changes were detected, the same as for the molecular weight.

Concerning the crystallinity degree (Table S1), the values of PBS treated with cutinase, calculated by DSC analysis, slightly increase. Shi et al. [20] observed a similar behaviour in PBS degraded by cutinase, ascribing the increase to the rearrangement of degraded amorphous chains that have more space to move and thus to form new organized domains. On the other hand, the crystallinity degree of PBSA, PCL6500D and PCL6900D films after enzymatic degradation did not show a particular trend, matching the value of pristine film (Table S1).

In summary, all results support what is stated above: the degradation occurs by surface erosion, and the molecular and thermal properties of the solid residue films did not significantly change.

#### 3.3.2. Liquid Fraction Characterization: Organic Extraction

The liquid fraction deriving from degradation experiments was subjected to a number of analyses, i.e., GPC and ^1^H NMR after its extraction in CH_2_Cl_2_, in order to evaluate both residual solvent-soluble oligomers and some monomers, and by HPLC-RID, in order to identify and quantify the water-soluble monomers (see Section 3.3.3). These analyses are useful to understand the prevalent cleaving action mode of each enzyme on the different polyesters. GPC results performed on the last liquid fraction of selected samples highlighted the presence of low molecular weight species (Table 6). Considering the PBS treated with cutinase, oligomers with M_w_ corresponding to dimers (the monomeric unit of PBS is 172 g/mol, thus the dimer is 344 g/mol) were supposed to be mainly released in the liquid fraction. Conversely, higher M_w_ oligomers, up to trimers and tetramers (monomeric unit of PBSA 180 g/mol; dimer 360 g/mol; trimer 540 g/mol; tetramer 720 g/mol) were identified in the liquid fraction of PBSA after incubation with lipases from *Pseudomonas fluorescens* and *Alcaligenes* sp. (QLM), respectively. The same consideration can be taken for PCL. Since the monomeric unit of this polyester is 114 g/mol, we can speculate that oligomers were mainly constituted by trimers (342 g/mol) and tetramers (456 g/mol) in all samples (possibly octamers, 912 g/mol, in case of Lipase CALB). Of course, low M_w_ molecules could also be originated from further specific degradation products.

Concerning ^1^H NMR analysis, in ESI the spectrum of the treated samples was compared to the pristine polymer and the monomers, for more clarity. The main conclusions from the results are reported in Table 6. In the case of PBSA, the signals of BD and oligomers were always found (ESI, Figure S2c–e), even if in the presence of cutinase the signals ascribable to the oligomers had very low intensity, suggesting that this enzyme was able to depolymerize PBSA mainly into the respective monomers. Moreover, the ratio BA/BS increased in samples treated with lipases from *Pseudomonas fluorescens* and *Alcaligenes* sp. (QLM), indicating that more adipic moieties were released in the liquid fraction of these samples. Concerning PCL6500D samples treated with cutinase and lipase from *Candida* sp. (CALB), the ^1^H NMR spectra displayed only HA (ESI, Figure S3c,d), while the samples treated with the lipases from *Pseudomonas fluorescens* and *Alcaligenes* sp. (QLM) exhibited oligomers in addition to HA (ESI, Figure S3e,f). These results would indicate that cutinase and CALB lipase were able to hydrolyze PCL6500D mainly into an HA monomer. Concerning PCL6800D, the spectra always showed the presence of both HA and oligomers, even though in the case of cutinase the signals ascribable to oligomers have a very low intensity, confirming as expected its higher monomerization activity also on PCL6800D (ESI, Figure S3h,i).

#### 3.3.3. Liquid Fraction Characterization: Water-Soluble Monomers

**Figure 5:**
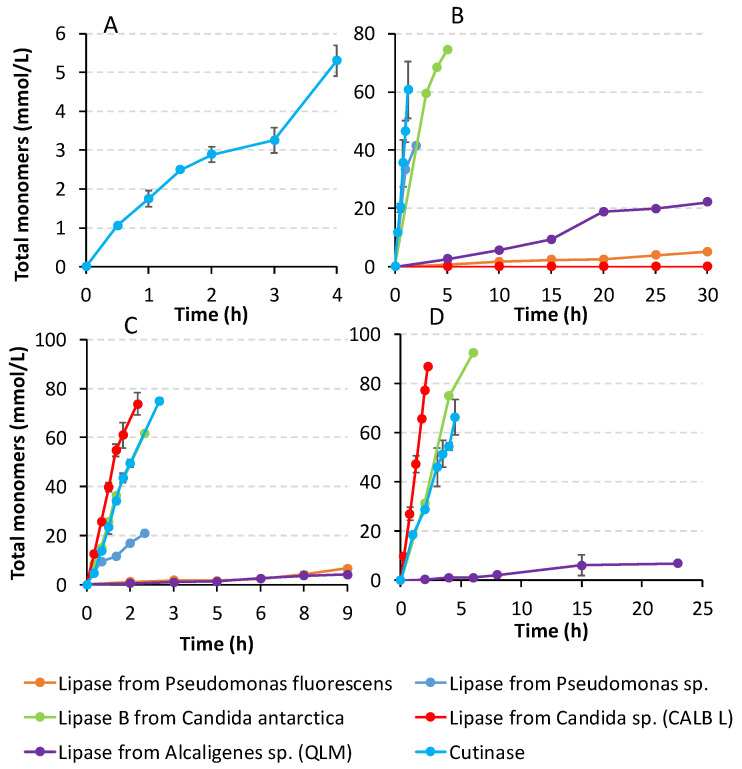
Concentration of the total monomers (mmol/L) detected via HPLC-RID analysis in the liquid fraction of PBS (**A**), PBSA (**B**), PCL6500D (**C**) and PCL6800D (**D**) degraded samples by the different enzymes over time (hours).

**Figure 6:**
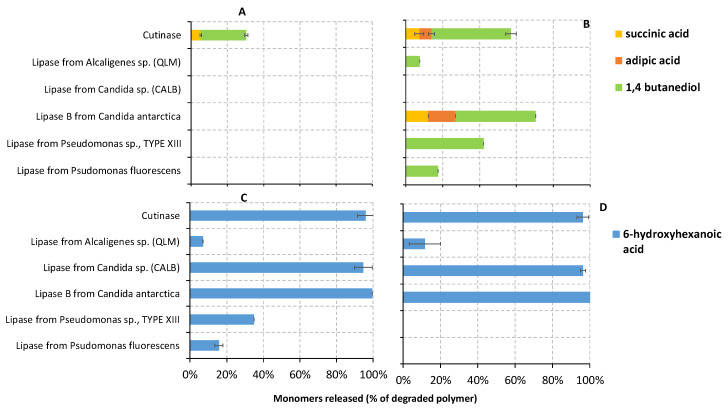
A fraction (%) of the degraded PBS (**A**), PBSA (**B**), PCL6500D (**C**), and PCL6800D (**D**) films which have been hydrolyzed into monomers by several enzymes in the last sampling time.

The water-soluble monomers released during the enzymatic degradation of the polyesters (PBS, PBSA, PCL6500D and PCL6800D) in the liquid fraction were also identified and quantified via HPLC-RID at each sampling time (Figure 5). The percentage of the degraded polymers fully hydrolyzed into each monomer by the different enzymes was also determined for all sampling times, in order to obtain a more quantitative point of view. Figure 6 shows the percentage calculated only for the last sampling time.

No monomers were detected in the negative enzyme-free controls (data not shown), according to the negligible or absent abiotic hydrolysis occurring on polyesters films when incubated in a buffer solution without enzymes. Conversely, the total monomer concentration (mmol/L) of PBS (SA and BD), PBSA (SA, AA and BD), PCL6500D and PCL6800D (HA) samples treated with the different enzymes tends to increase linearly over time (Figure 5). Actually, the weight loss of PBS treated with cutinase (approximately 63%) at the end of incubation was much greater than the total monomer concentration quantified in the last liquid fraction, corresponding to approximately 30.5% of the degraded PBS (Figure 6). Therefore, it can be assumed that PBS weight loss was initially caused by a release of many oligomers, which were further hydrolyzed into monomers as polymer degradation proceeded. This agrees with GPC data, which revealed the presence of dimers in the liquid fraction. Based on these results, a prevalent endo-type mechanism might be suggested for the hydrolysis of PBS by cutinase. Moreover, the concentration of BD was higher than that of SA (Figure S9) at each sampling time; for example, the fraction of the degraded polymer that was hydrolyzed into BD and SA corresponded to approximately 25 and 5%, respectively, after 4 h incubation (Figure 6A). This would indicate that cutinase cuts the polyester chain mainly from the OH-ends. Shi et al. (2019) [20] also suggested an endo-type mode of action for cutinase on PBS, since they observed SA monomer and short chain oligomers as the main degradation products; however, they did not find BD monomer during enzymatic degradation, indicating that cutinase cuts the polymer chain from the carboxyl end. On the contrary, Lee et al. [19] proposed an exo-type mechanism for the hydrolysis of PBS by lipase from *Pseudomonas cepacia,* since 4-hydroxybutyl succinate dimer was detected as the main degradation product. Li et al. [67] also revealed succinic acid and succinates rather than PBS oligomers and BD monomers as PBS degradation products, indicating an exo-type action mode and a carboxyl end cleaving for the PBS-degrading enzyme from *Aspergillus* sp.

In the case of PBSA, no monomers were detected in the samples treated with lipase from *Candida* sp. (CALB), according to the negligible weight loss of PBSA film which occurred in this sample. Conversely, faster and greater total monomer concentration was detected in the samples treated with cutinase, lipase from *Pseudomonas* sp. and lipase B from *Candida antarctica* (Figure 5B), where higher enzymatic degradation rates also occurred. Looking at the different monomers, PBSA samples treated with lipase B from *Candida antarctica* and cutinase released over time all monomers, i.e., SA, AA, and BD. Finally, only BD was detected in the samples treated with all the other enzymes, i.e., lipases from *Pseudomonas fluorescens*, *Pseudomonas sp*., and *Alcaligenes* sp. (QLM) (Figure S9B,C,E, and Figure 6B). The release of BD alone is unlikely, suggesting that the dicarboxylic acids in these samples might be undetectable possibly due to concentrations below the detection limit. However, the greater concentration of the diol could be explained as previously described for the PBS. Lipase B from *Candida antarctica* showed the highest degree of depolymerization, fully hydrolyzing more than 70% of the degraded polymer into three monomers (12.3, 14.8 and 43.5% of SA, AA and BD, respectively) at the end of incubation (Figure 6B). Similar depolymerization occurred with cutinase, hydrolyzing almost 60% of PBSA into monomers (7.3, 6.6 and 43.0% of SA, AA and BD, respectively). Given the higher PBSA monomerization and the lower amount of oligomers revealed by the ^1^H NMR analyses, it could be speculated that cutinase acted mainly as an exo-type enzyme, although some endo-type scission is exerted as indicated by the fact that released monomers account for about 60% of the degraded polymer mass. This same prevalent mechanism might be also proposed for lipase B from *Candida antarctica* because of the high percentage of PBSA depolymerization revealed by HPLC analyses. Conversely, lipases from *Pseudomonas fluorescens and Alcaligenes* sp. (QLM) revealed a lower degree of monomerization, hydrolyzing only 17.4 and 7.6% of PBSA into BD monomer, respectively (Figure 6B). In the last cases, polymer films were mainly hydrolyzed in the low molecular weight oligomers identified by GPC analyses (Table 5). Therefore, an endo-type action could be suggested for these enzymes given both the lower monomerization (revealed by HPLC results) and the greater amount of oligomers (revealed by ^1^H NMR). Lipase from *Pseudomonas sp.*, which hydrolyzed approximately 40% of PBSA into monomers (Figure 6B), could be also identified as endo-type enzymes. Compared to the SA monomer, a higher concentration of AA was mainly detected as degradation product of lipase B from *Candida antarctica* and cutinase-treated polymers, although the ratio of AA in PBSA copolymer is much less than that of other acid monomer (AA:SA:BD is 13:37:50% mol/mol). The results presented here suggested, therefore, that these enzymes preferentially hydrolyzed the adipic acid segments rather than the succinic acid ones. This could be explained by the longer chain of AA (C6) compared to that of SA (C4). It is well demonstrated that the enzymatic degradation of polyesters strongly depends on the distance between ester groups, as hydrolysis occurs preferentially at the ester groups with greater methylene contents [12].

Concerning PCL polyesters, an HA monomer was detected in the liquid fraction of all samples. However, a faster and higher release of HA occurred in both PCL6500D (Figure 5C) and PCL6800D samples (Figure 5D) treated with lipase from *Candida* sp. (CALB), lipase B from *Candida antarctica* and cutinase, where greater PCL degradative activity was also observed during weight loss tests. Moreover, the percentage of degraded polymers hydrolyzed into HA was almost 100% in the PCL samples treated with lipase B from *Candida antarctica*, lipase CALB and cutinase (Figure 6C,D), suggesting that these enzymes were able to complete depolymerize PCL into monomers. These results are consistent with the ^1^H NMR data, where only HA was detected in the presence of cutinase and CALB. Therefore, we can speculate an exo-type degradation mechanism of PCL for these enzymes, i.e., lipase B from *Candida Antarctica*, lipase CALB and cutinase. On the contrary, lipase from *Alcaligenes* sp. (QLM) was not able to completely depolymerize PCL films into monomers, since approximately 7 and 17% of PCL6500D and PCL6800D were hydrolyzed into HA, respectively (Figure 6C,D). Similarly, lipases from *Pseudomonas fluorescens* and *Pseudomonas* sp. did not fully depolymerize PCL6500D into monomers (Figure 6C). These results agree with the ^1^H NMR data, which reported a higher signal of oligomers in PCL6500D samples treated with both *Pseudomonas fluorescens* and QLM lipase, as well as in PCL6800D samples treated with QLM lipase. The higher presence of oligomers and the lower monomerization suggest an endo-type PCL degradation mechanism for lipases from *Pseudomonas fluorescens*, *Pseudomonas* sp. and QLM.

In summary, PBS is hydrolyzed by cutinase by endo-type, producing mostly oligomers. PBSA films were predominantly hydrolyzed into oligomers, whereas both PCL films into monomers by most of enzymes. Hoshino and Isono [4] also observed that PBS and PBSA were mainly degraded to dimers, while PCL to monomers by three different commercial lipases, namely lipase Asahi from *Chromobacterium viscosum,* lipase F from *Rhizopus niveus*, and lipase F-AP15 from *Rhizopus orizae*. Complete PCL hydrolysis into monomers by a bifunctional lipase-cutinase expressed in *Pichia pastoris* was also observed in the study conducted by Liu et al. [35], where a large amount of HA and only traces of PCL oligomers were observed as degradation products.

Moreover, lipases from *Pseudomonas fluorescens*, *Pseudomonas* sp. and *Alcaligenes* sp. (QLM) exhibited the lowest depolymerization into monomers in all polymer samples (Figure 6). It is observed that some lipases require hydrophobic interaction with substrates for their activation [21], so, possibly, less hydrophobic oligomers such as dimers, trimers and tetramers were not easily hydrolyzed into monomers by these enzymes. The ability of some *Pseudomonas* lipase to degrade polymer into short oligomers but not monomers were also reported by previous studies [68].

## 4. Conclusions

In this study, several commercial hydrolytic enzymes (several lipases, proteinase K, cutinase) were investigated for their ability to degrade different aliphatic biopolyesters, i.e., PBS, PBSA, two PCL having lower (PCL6500D) and higher (PCL6800D) molecular weight, PLA and PPC. In the preliminary screening, PBSA and both PCL polyesters were completely hydrolyzed by several enzymes in a few hours, whereas PBS homopolymer was hydrolyzed only by the cutinase. None of the tested enzymes were able to completely hydrolyze both PLA and PPC films in the conditions employed in this study. Cutinase showed the highest degradation rate (6.4 mg/h/cm^2^) against PBSA copolymer, whereas lipase from *Candida* sp. (CALB) against PCL6500D (5.2 mg/h/cm^2^). Lower degradation rates were observed for higher molecular weight PCL (PCL6800D). Chemical analyses performed on the selected PBS, PBSA, PCL6500D and PCL6800D films treated with different enzymes showed that enzymatic degradation mechanism proceeded by degrading the polymer chains from the surface of the film. The thermal properties of degraded polyesters did not show any significant differences in all polymers. Cleaving action mode for each enzyme (endo- and/or exo-type) against PBS, PBSA, PCL6500D and PCL6800D were also proposed based on the identification and quantification of the oligomers and monomers released as degradation products in the liquid fraction. Generally, PBS and PBSA films were enzymatically hydrolyzed mainly into oligomers, although higher monomerization activity against PBSA copolymer was exhibited by lipase B from *Candida antarctica* and cutinase. Conversely, many enzymes (lipase B from *Candida antarctica*, lipase CALB and cutinase) were able to almost completely depolymerize into monomers both PCL polyesters. The high degradation rates achieved by several enzymes on PBS, PBSA and PCL films, together with the deep investigation of their hydrolysis mechanisms in this study, and contribute significantly to clarify the enzymatic degradation of aliphatic polyesters. This could be useful for the management of biodegradable plastic wastes, the bioremediation of plastic-polluted environments, as well as the design of innovative biodegradable plastic materials.

## Figures and Tables

**Table 1:** Hydrolytic enzymes with indication of the supplier: A, Sigma-Aldrich; B, ChiralVision; C, J&K, and conditions used in the preliminary screening and weight loss test, such as polymers tested, pH, temperature (°C) and enzyme concentration (U/mL).

Enzyme	Supplier	General Screening Conditions			Weight Loss Test Conditions		
		Tested Polymer	pH; T (°C)	Enzyme Concentration (U/mL)	Tested Polymer	pH; T (°C)	Enzyme Concentration (U/mL)
Amano Lipase from *Pseudomonas fluorescens*	A	PBS; PBSA; PCL6500D; PCL6800D; PLA; PPC	8; 40	50	PBSA; PCL6500D	8; 40	10
Lipase from *Pseudomonas* sp., TYPE XIII	A	PBS; PBSA; PCL6500D; PCL6800D; PLA; PPC	7; 40	10	PBSA; PCL6500D	7; 40	10
Lipase from *Rhizopus niveus*	A	PBS; PBSA; PCL6500D; PCL6800D; PLA; PPC	7; 40	100	Not tested		
Lipase from *Rhizopus oryzae* (Lipase F-AP 15), LIGHT B	A	PBS; PBSA; PCL6500D; PCL6800D; PLA; PPC	7; 40	100	PCL6500D	7; 40	10
Lipase B from *Candida antarctica*; recombinant from *Aspergillus oryzae*	A	PBS; PBSA; PCL6500D; PCL6800D; PLA; PPC	7; 40	10	PBSA; PCL6500D; PCL6800D	7; 40	10
Lipase from *Candida* sp., recombinant from *Aspergillus niger* (NZ CALB L)	A	PBS; PBSA; PCL6500D; PCL6800D; PLA; PPC	8; 40	100	PBSA; PCL6500D; PCL6800D	8; 40	10
Lipase from *Candida rugosa,* Type VII	A	PBS; PBSA; PCL6500D; PCL6800D; PLA; PPC	7; 40	100	Not tested		
Lipase from *Candida rugosa*	A	PBS; PBSA; PCL6500D; PCL6800D; PLA; PPC	8; 40	100	Not tested		
Lipase from *Candida cylindracea*	A	PBS; PBSA; PCL6500D; PCL6800D; PLA; PPC	7; 30	50	Not tested		
Lipase from hog pancreas	A	PBS; PBSA; PCL6500D; PCL6800D; PLA; PPC	8; 40	100	Not tested		
Lipase from *Alcaligenes* sp. (QLM)	B	PBS; PBSA; PCL6500D; PCL6800D; PLA; PPC	8; 40	100	PBSA; PCL6500D; PCL6800D	8; 40	10
Cutinase (NZ 51032)	B	PBS; PBSA; PCL6500D; PCL6800D; PLA; PPC	8; 40	100	PBS; PBSA; PCL6500D; PCL6800D	8; 40	10
Proteinasi K from *Tritirachium album*	C	PBS; PBSA; PCL6500D; PCL6800D; PLA; PPC	8; 40	100	Not tested		

**Table 2:** Molecular and thermal properties of polyester pellets as received.

	GPC		DSC						
			Cooling Scan		2nd Heating Scan				
Sample	M_w_ (×10^−3^ g/mol)	PD	T_c_ (°C)	ΔH_c_ (J/g)	T_g_ (°C)	T_cc_ (°C)	ΔH_cc_ (J/g)	T_m_ (°C)	ΔH_m_ (J/g)
PBS	186	2.5	63	50	−31	/	/	116	53
PBSA	195	2.4	41	38	−45	/	/	87	34
PCL6500D	153	1.5	21	49	−61	/	/	58	45
PCL6800D	216	1.5	14	50	nd	/	/	57	47
PLA	202	2.0	/	/	59	116	25	152	24
PPC	184	3.1	/	/	17	/	/	/	/

nd: not detectable in the conditions tested.

**Table 3:** Main results of the 24 h degradation test performed on the different polyesters (PBS, PBSA, PCL6500D, PCL6800D, PLA and PPC) with the different enzymes. * indicates that the degradation of the polymer film has not occurred; 100% indicates a complete degradation of the polymer occurred in the time indicated between parentheses.

Enzyme	PBS	PBSA	PCL 6500D	PCL 6800D	PLA	PPC
Amano Lipase from *Pseudomonas fluorescens*	*	100% (>24 h)	100% (6–24 h)	*	*	*
Lipase from *Pseudomonas sp*.	*	100% (4–6 h)	100% (2–4 h)	*	*	*
Lipase from *Rhizopus niveus*	*	*	*	*	*	*
Lipase from *Rhizopus oryzae*	*	*	100% (6–24 h)	*	*	*
Lipase B from *Candida antarctica*	*	100% (>24 h)	100% (1–2 h)	100% (6–24 h)	*	*
Lipase from *Candida* sp. (CALB)	*	100% (6–24 h)	100% (0.3–1 h)	100% (2–4 h)	*	*
Lipase from *Candida rugosa*, Type VII	*	*	*	*	*	*
Lipase from *Candida rugosa*	*	*	*	*	*	*
Lipase from *Candida cylindracea*	*	*	*	*	*	*
Lipase from hog pancreas	*	*	*	*	*	*
Lipase from *Alcaligenes* sp. (QLM)	*	100% (1–2 h)	100% (1–2 h)	100 (6–24 h)	*	*
Cutinase	100% (2–4 h)	100% (1–2 h)	100% (1–2 h)	100% (4–6 h)	*	*
Proteinasi K	*	*	*	*	*	*

**Table 4:** Enzymatic degradation rate (mg/h/cm^2^) of PBS, PBSA, PCL6500D and PCL6800D in the presence of the different enzymes.

Enzyme (10 U/mL)	PBS (mg/h/cm^2^)	PBSA (mg/h/cm^2^)	PCL6500D (mg/h/cm^2^)	PCL6800D (mg/h/cm^2^)
Lipase from *Pseudomonas fluorescens*	Not tested	0.10 ± 0.01	0.54 ± 0.01	Not tested
Lipase from *Pseudomonas sp.*	Not tested	1.91	3.13	Not tested
Lipase B from *Candida antarctica*	Not tested	1.17	3.52	1.41
Lipase from *Candida* sp. (CALB)	Not tested	0.00 ± 0.00	5.17 ± 0.51	4.78 ± 0.30
Lipase from *Alcaligenes* sp. (QLM)	Not tested	0.16 ± 0.00	0.72 ± 0.02	0.33 ± 0.03
Cutinase	0.35 ± 0.01	6.38 ± 0.66	4.57 ± 0.05	1.96 ± 0.15

**Table 5:** ^1^H NMR and GPC of PBS, PBSA, PCL6500D and PCL6800D residual solids after treatment with different enzymes.

				^1^H NMR	GPC		
Sample	Enzyme	Time (h)	Wt Loss (%)	OH End-Groups (mol%)	M_n_ (×10^−3^ g/mol)	M_w_ (×10^−3^ g/mol)	PD
PBS	Control	30	/	1.1	74	186	2.5
	Cutinase	1	16	1.7	73	178	2.4
		2	30	1.5	70	173	2.5
		4	63	1.8	68	176	2.6
PBSA	Control	30	/	1.1	81	195	2.4
	Cutinase	0.5	45	1.6	67	164	2.5
		1	80	2.2	72	181	2.6
	Lipase from *P. fluorescens*	15	11	1.6	62	163	2.6
		25	24	1.9	61	157	2.6
		30	29	1.8	61	155	2.5
	Lipase from *Alcaligenes* sp. (QLM)	10	29	1.6	63	160	2.6
		20	45	2.1	62	162	2.6
		30	61	1.9	60	164	2.7
	Lipase from *Pseudomonas sp.*	1	60	na	64	160	2.5
		2	94	na	61	152	2.5
	Lipase B from *C. antarctica*	3	68	na	64	164	2.6
		4	78	na	62	152	2.5
		5	93	na	57	155	2.7
PCL 6500D	Control	30	/	0.5	102	153	1.5
	Cutinase	0.5	23	0.8	87	133	1.6
		1	48	1.2	90	141	1.6
		1.5	68	1.6	81	130	1.6
	Lipase from *Candida* sp. (CALB)	0.3	21	0.6	95	146	1.5
		0.8	53	0.6	91	138	1.5
		1.3	74	1.2	90	140	1.6
	Lipase from *P. fluorescens*	3	16	0.6	94	146	1.5
		6	32	1.4	87	138	1.6
		9	58	1.3	88	142	1.6
	Lipase from *Alcaligenes* sp. (QLM)	3	23	1.2	80	122	1.5
		6	45	1.4	78	123	1.6
		9	66	1.7	78	118	1.5
	Lipase from *Pseudomonas* sp.	0.5	27	na	79	125	1.6
		1.5	57	na	80	124	1.5
		2	79	na	80	123	1.5
	Lipase B from *C. antarctica*	0.5	23	na	77	119	1.5
		0.8	40	na	85	129	1.5
		1	55	na	82	127	1.5
		2	92	na	75	118	1.6
PCL 6800D	Control	30	/	1.0	120	197	1.64
	Cutinase	1	24	1.3	115	189	1.64
		3	61	1.0	114	185	1.63
		4	89	1.2	121	197	1.63
	Lipase from *Candida* sp. (CALB)	0.8	32	1.4	110	180	1.63
		1.8	67	1.4	115	189	1.65
		2	88	1.2	123	188	1.53
	Lipase from *Alcaligenes* sp. (QLM)	4	14	1.2	102	170	1.67
		8	32	1.2	102	172	1.68
		15	65	1.2	107	183	1.71
	Lipase B from *C. antarctica*	2	23	na	99	172	1.74
		4	66	na	91	153	1.69
		6	93	na	94	161	1.72

na: not available.

**Table 6:** ^1^H NMR and GPC of the products extracted from the last liquid fraction of PBS, PBSA, PCL6500D and PCL 6800D films after treatment with different enzymes (BA/BS ↑ indicates that the adipate content increases respect to the succinate content).

Polymer	Enzyme			GPC		^1^H NMR	Prevalent Cleaving Action Mode *
		Time (h)	Wt. Loss (%)	M_w_ (g/mol)	PD	Notes	
PBS	Cutinase	4	63	383	1.2	na	Endo-type scission
PBSA	Cutinase	1.3	99	na	na	Oligomers traces + BD	Exo-type scission
	Lipase from *Pseudomonas fluorescens*	30	29	484	1.3	Oligomers + BD BA/BS ↑	Endo-type scission
	Lipase from *Alcaligenes* sp. (QLM)	30	61	646	1.4	Oligomers + BD BA/BS ↑	Endo-type scission
PCL6500D	Cutinase	2.5	98	431	1.3	HA	Exo-type scission
	Lipase from *Candida* sp. (CALB)	1.8	93	428	1.3	HA	Exo-type scission
	Lipase from *Pseudomonas fluorescens*	9	59	306	1.1	Oligomers + HA	Endo-type scission
	Lipase from *Alcaligenes* sp. (QLM)	9	66	297	1.2	Oligomers + HA	Endo-type scission
PCL6800D	Cutinase	4.5	100	370	1.2	Oligomers traces + HA	Exo-type scission
	Lipase from *Candida* sp. (CALB)	2.3	93	982	1.5	Oligomers + HA	Exo-type scission
	Lipase from *Alcaligenes* sp. (QLM)	23	96	256	1.5	Oligomers +HA	Endo-type scission

* proposed on the basis also of the analysis of the water-soluble monomers (Section 3.3.3). na: not available.

## Data Availability

All data are contained in the ESI or available upon email request from the authors.

## Supplementary Materials

The following supporting information can be downloaded at: https://www.mdpi.com/article/10.3390/polym14091850/s1, Figures S1–S5. NMR spectra. Figure S6. Calorimetric curves of polyesters. Figure S7a,b. Thermogravimetric curves and dTGA curves of polyesters. Figure S8. ATR FT-IR curves of polyester residual solids: PBSA after treatment with *Pseudomonas fluorescens* (A) and QLM (B), PCL 6500D after treatment with *Pseudomonas fluorescens* (C), CALB (D) and QLM (E), and PCL 6800D after treatment with CALB (F) and QLM (G). Figure S9. Concentration of succinic acid and 1,4 butanediol (mmol/L) detected via HPLC-RID analysis in the liquid fraction of PBS sample degraded by cutinase (A) over time (hours). Concentration of succinic acid, adipic acid and 1,4 butanediol (mmol/L) detected via HPLC-RID analysis in the liquid fraction of PBSA samples degraded by lipase from *Pseudomonas fluorescence* (B), lipase from *Pseudomonas* sp. (C), lipase B from *Candida antarctica* (D), lipase from *Alcaligenes sp.,* QLM (E) and cutinase (F) over time (hours). Table S1. DSC results of PBSA, PBSA, PCL6500D and PCL6800D residual solids after treatment with different enzymes. Table S2. Regiosequences of PPC on a diad level and on a tetrad level [69].
