# Supplementary material for: Enzymatic Degradation of the Most Common Aliphatic Bio-Polyesters and Evaluation of the Mechanisms Involved: An Extended Study
Source: Polymers (Basel). 2022 Apr 30;14(9):1850. doi: 10.3390/polym14091850 (PMC9101158; doi:10.3390/polym14091850)
Supplement: Supplementary file 1 [file polymers-14-01850-s001.zip › polymers-1663351-supplementary.pdf]

Enzymatic degradation of the most common aliphatic biopolyesters and evaluation of the mechanisms involved: an extended study

Antonella Rosato, Angela Romano, Grazia Totaro, Annamaria Celli, Fabio Fava, Giulio Zanaroli*, Laura Sisti

Dept. of Civil, Chemical, Environmental and Material Engineering (DICAM), *Alma Mater Studiorum* University of Bologna, Via Terracini 28, 40131 Bologna, Italy

*corresponding author: giulio.zanaroli@unibo.it

Antonella Rosato and Angela Romano authors contributed equally to this work.

Table S1. DSC results of PBSA, PBSA, PCL6500D and PCL6800D residual solids after treatment with different enzymes.

				DSC					
				Cooling scan		Heating scan			
**Sample**	**Enzyme**	**Time (h)**	**Weight loss (%)**	T_c (°C)	ΔH_c (J/g)	T_g (°C)	T_m (°C)	ΔH_m (J/g)	X_c (%)
**PBS**	Control (enzyme-free)	30	/	93	65	-31	114	65	33
	Cutinase	1	16	93	63	-34	113	64	32
		2	30	93	68	-33	114	69	35
		4	63	93	74	-33	114	73	37
**PBSA**	Control (enzyme-free)	30	/	43	45	-45	86	46	32
	Cutinase	0.5	45	46	43	-48	85	45	32
		1	80	39	43	-48	85	45	32
	Lipase from *Pseudomonas fluorescens*	15	11	45	42	-46	87	43	30
		25	24	43	46	-46	87	45	32
		35	29	44	45	-46	86	46	32
	Lipase from *Alcaligenes sp.* (QLM)	10	29	41	43	-49	86	44	31
		20	45	40	46	-48	86	47	33
		30	61	41	45	-48	86	45	32
**PCL6500D**	Control (enzyme-free)	30	/	30	55	nd	56	57	41
	Cutinase	0.5	23	25	54	nd	56	54	39
		1	48	26	58	nd	55	58	42
		1.5	68	27	55	nd	56	57	41
	Lipase from *Candida sp.* (CALB)	0.25	21	28	53	nd	56	54	39
		0.75	53	28	53	nd	56	55	40
		1.25	74	28	53	nd	56	54	39
	Lipase from *P. fluorescens*	3	16	28	51	nd	57	51	37
		6	32	28	54	nd	57	54	39
		9	58	29	54	nd	57	54	39
	Lipase from *Alcaligenes sp.*(QLM)	3	23	31	54	nd	58	54	39
		6	45	31	56	nd	58	56	40
		9	66	31	53	nd	58	54	39
**PCL6800D**	Control (enzyme-free)	30	/	29	49	nd	56	52	37
	Cutinase	1	24	29	51	nd	56	55	40
		3	61	29	50	nd	56	52	37
		4	89	27	49	nd	56	51	35
	Lipase from *Candida sp.* (CALB)	0.75	32	29	49	nd	56	52	37
		1.75	67	29	51	nd	56	53	38
		2	88	29	49	nd	56	52	37
	Lipase from *Alcaligenes sp.* (QLM)	4	14	28	52	nd	56	51	37
		8	32	29	48	nd	56	49	35
		15	65	29	51	nd	56	50	36

na: not available

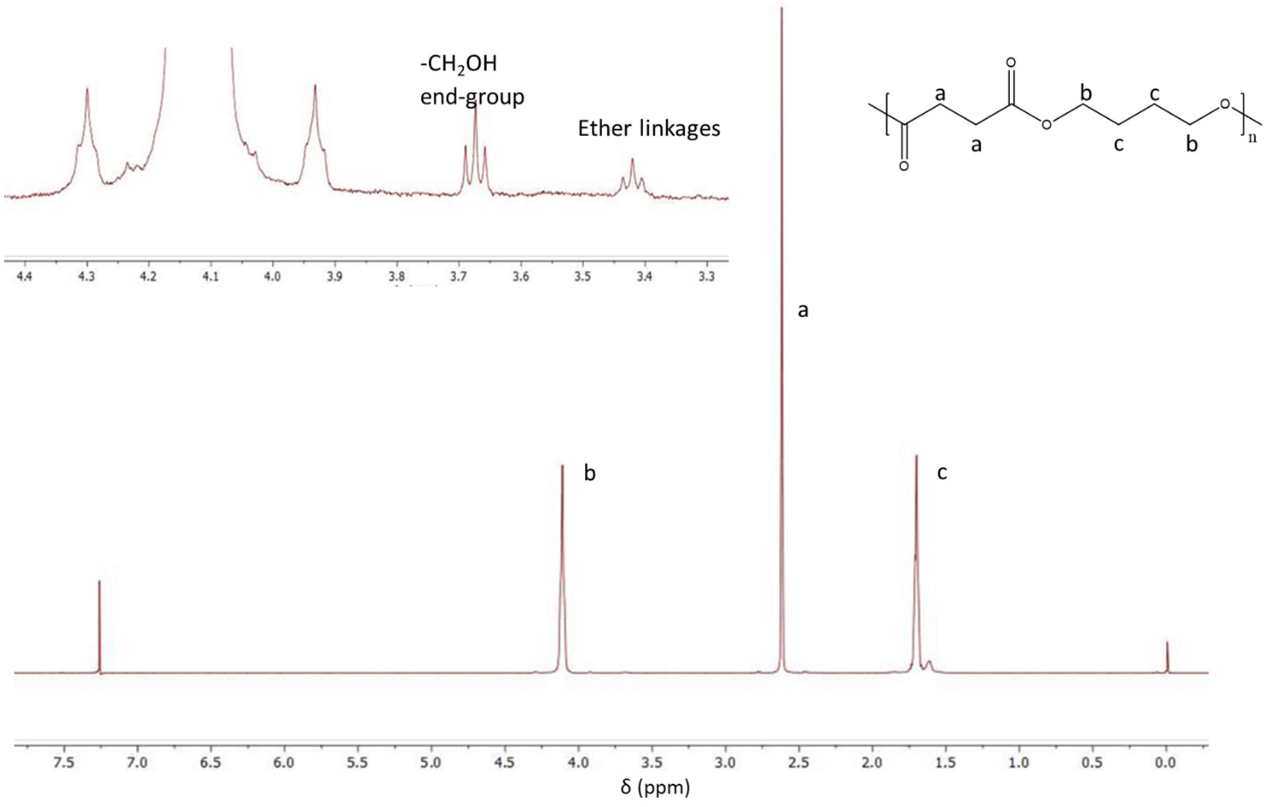

Figure S1a. ^{1}H NMR of PBS.

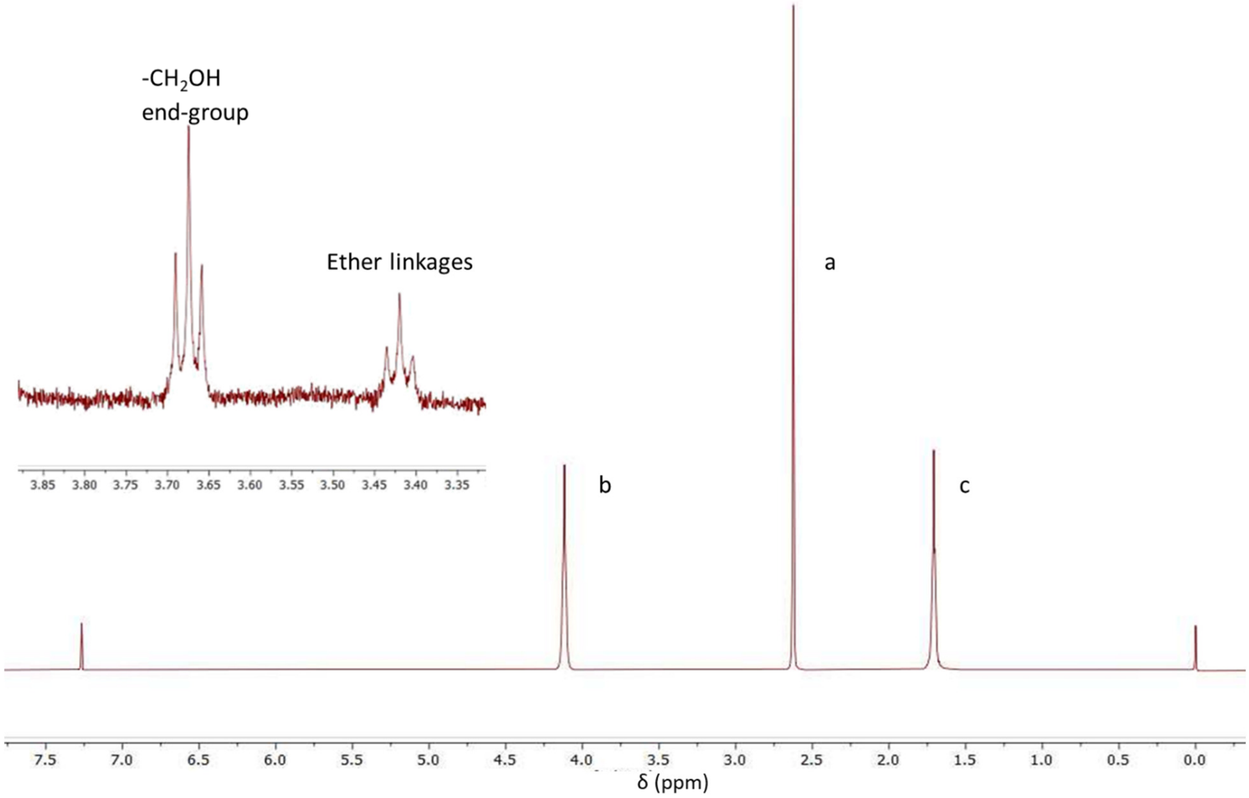

Figure S1b. ^{1}H NMR of PBS after incubation with cutinase (4 h).

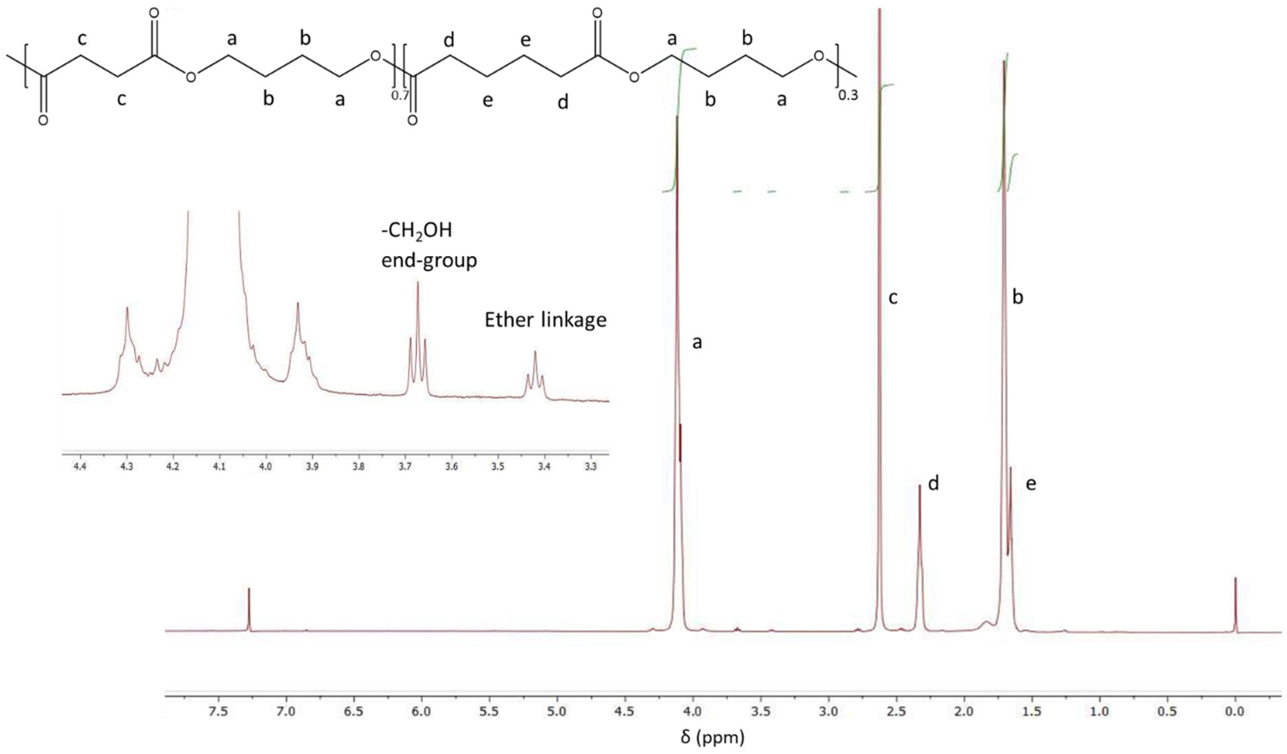

Figure S2a. ^{1}H NMR of PBSA.

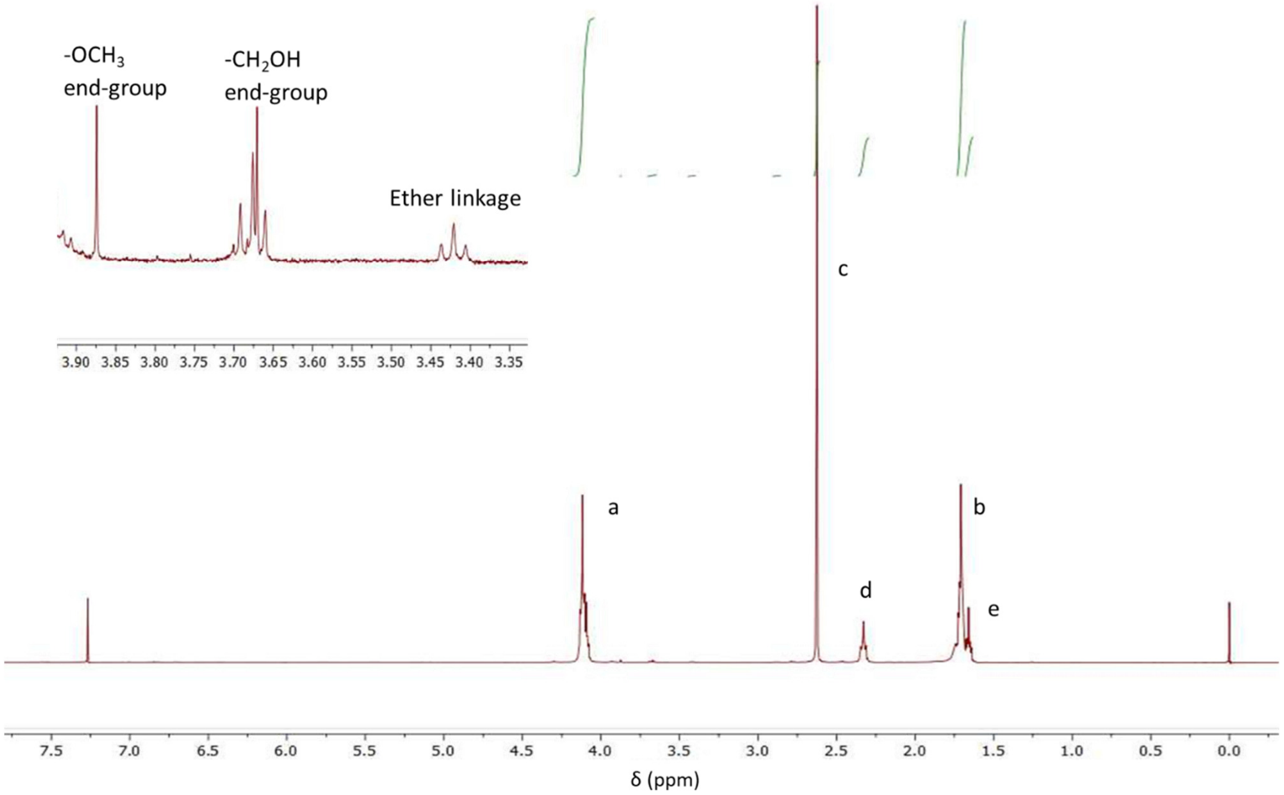

Figure S2b. ^{1}H NMR of PBSA after incubation with cutinase (1 h).

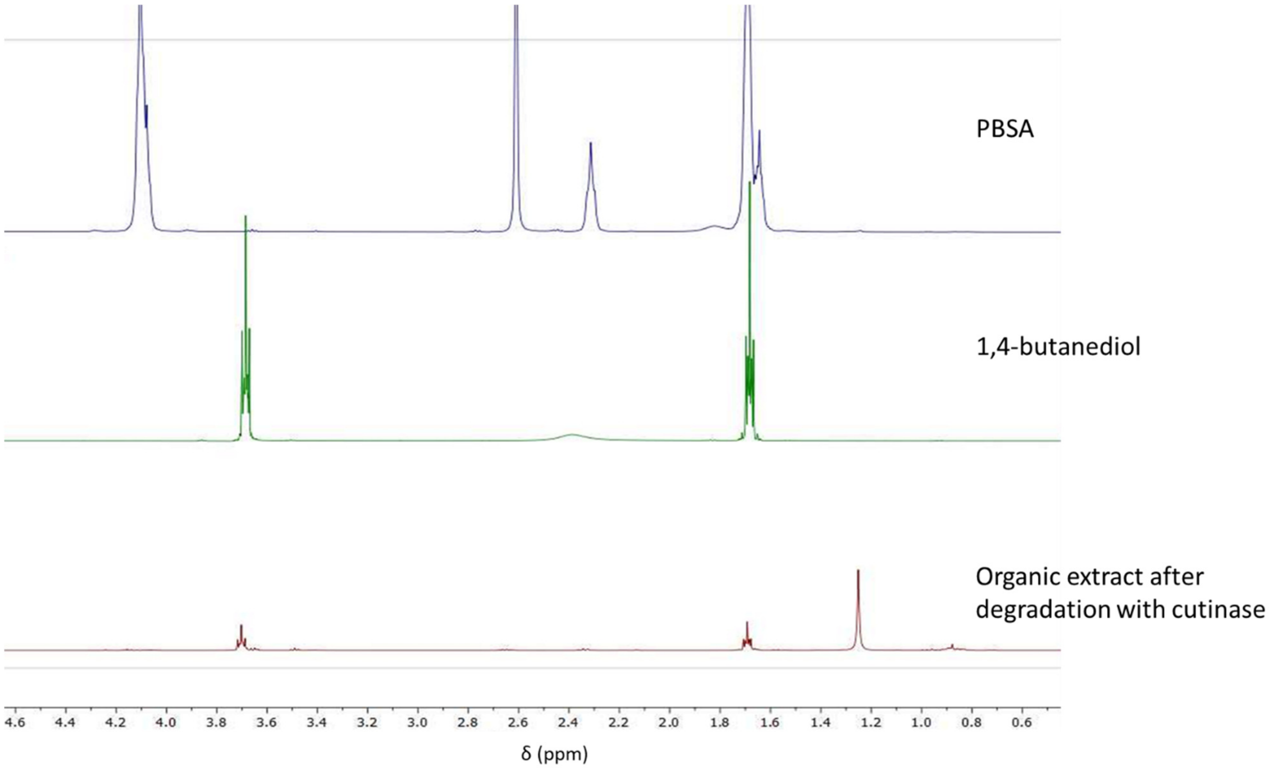

Figure S2c. ^{1}H NMR of the organic extract after degradation of PBSA with cutinase, compared to pristine PBSA and 1,4-butanediol.

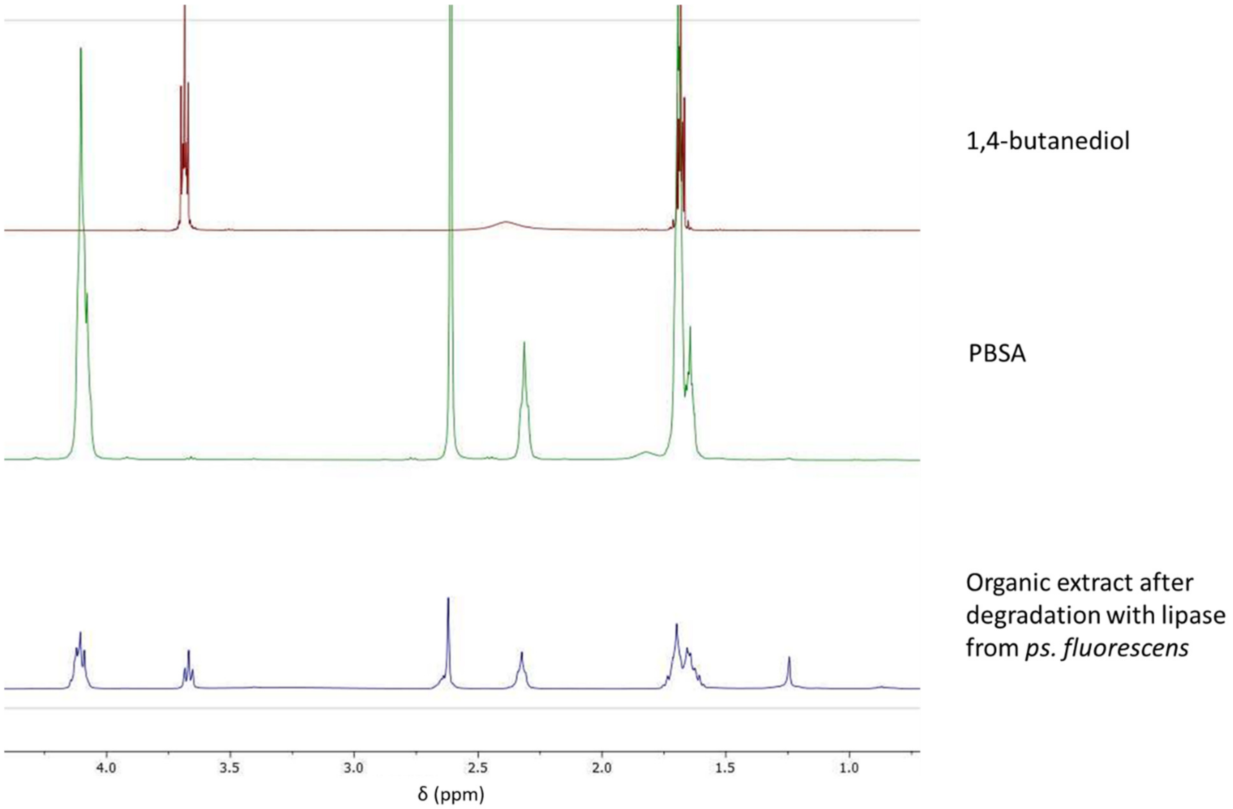

Figure S2d. ^{1}H NMR of the organic extract after degradation of PBSA with lipase from *ps. fluorescens*, compared to pristine PBSA and 1,4-butanediol.

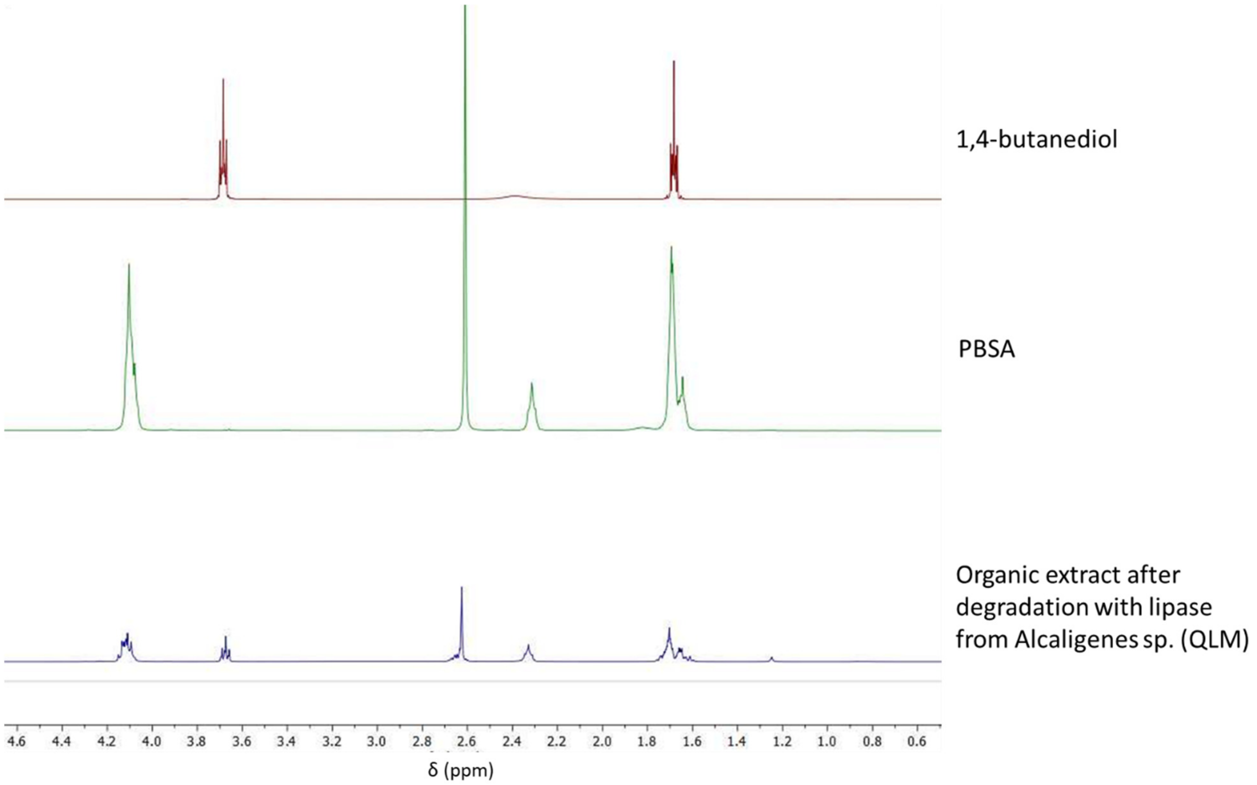

Figure S2e. ^{1}H NMR of the organic extract after degradation of PBSA with lipase from *Alcaligenes sp.* (QLM), compared to pristine PBSA and 1,4-butanediol.

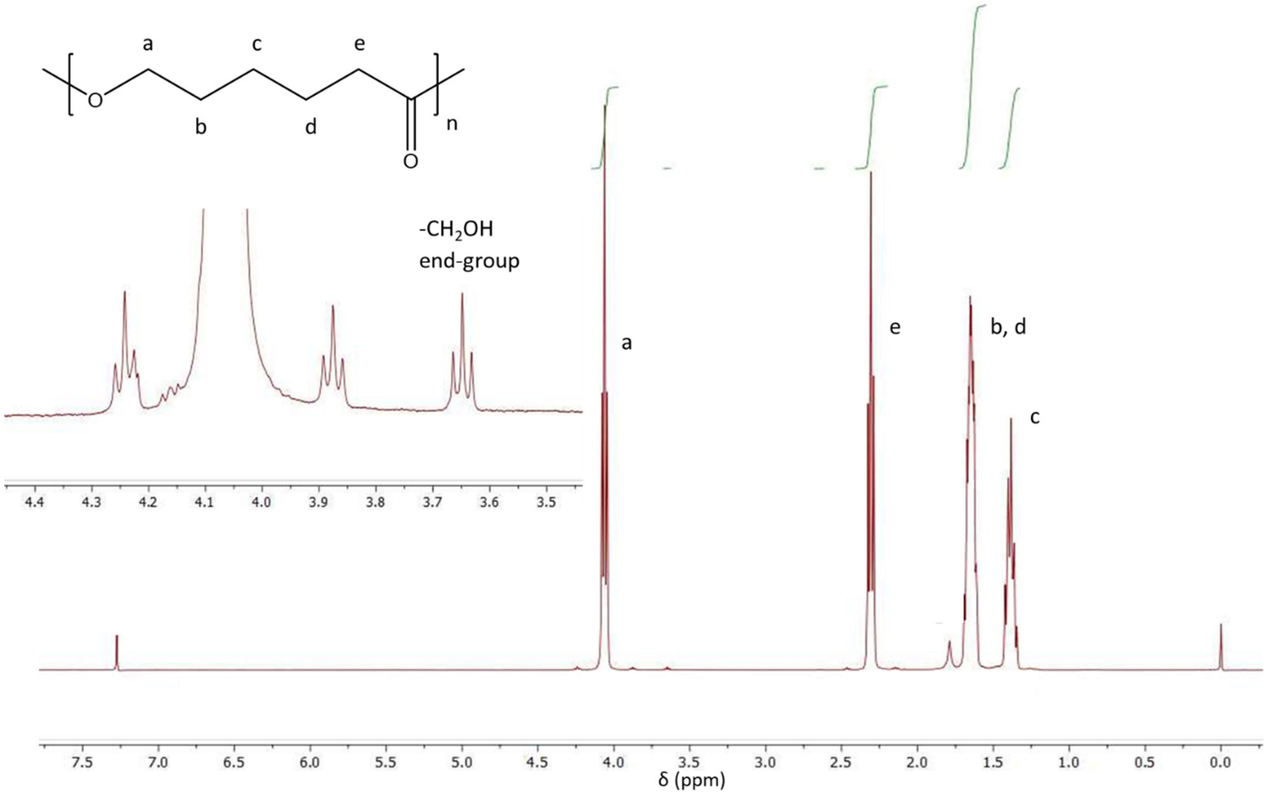

Figure S3a. ^{1}H NMR of PCL (6500D and 6800D).

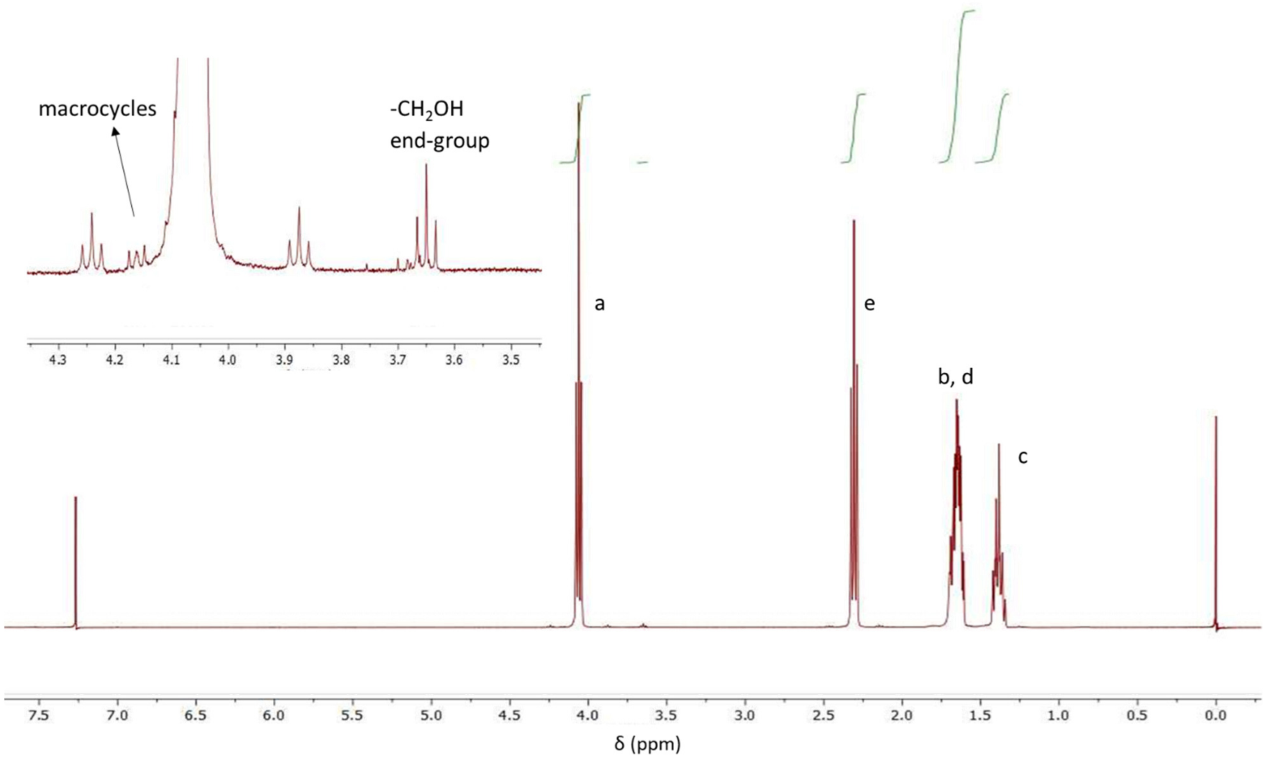

Figure S3b. ^{1}H NMR of PCL6500D after incubation with cutinase (1.5 h).

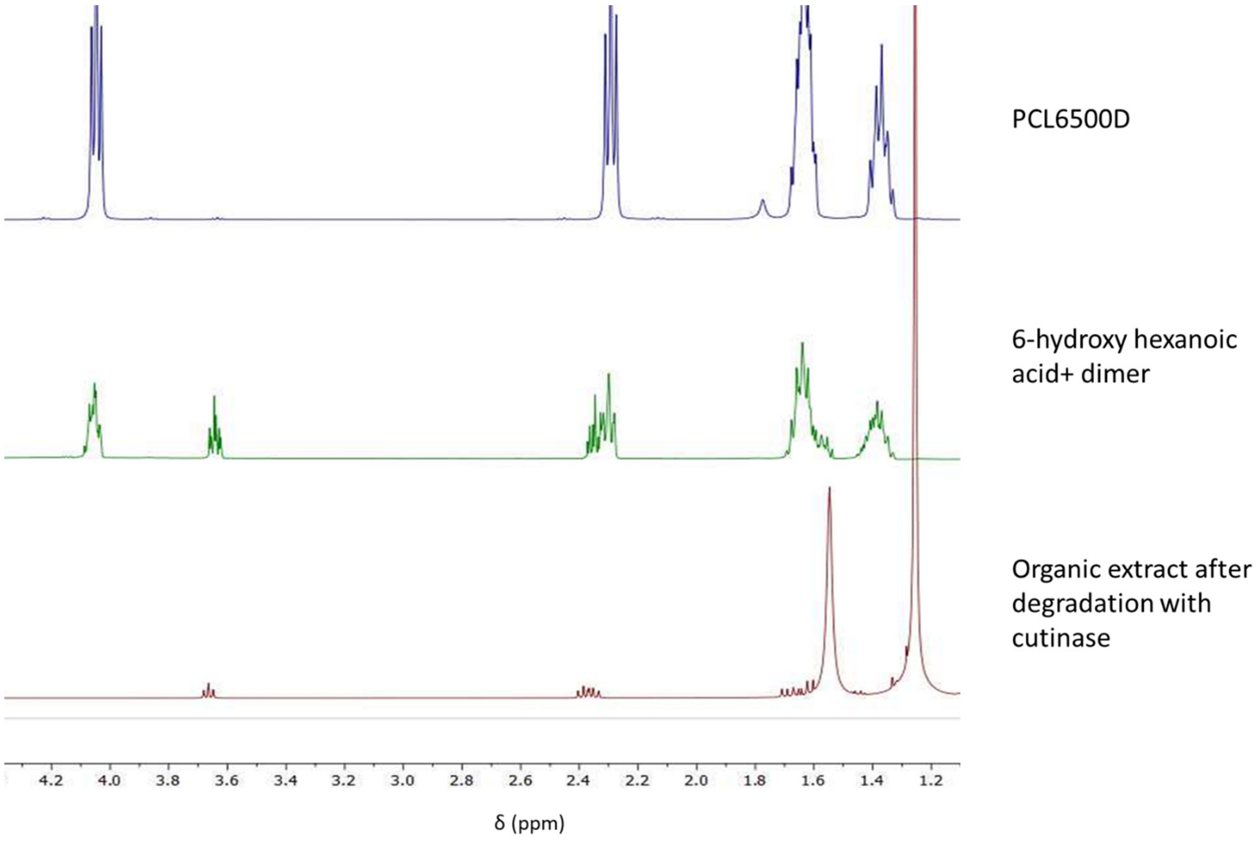

Figure S3c. ^{1}H NMR of the organic extract after degradation of PCL6500D with cutinase, compared to pristine PCL6500D and 6-hydroxyhexanoic acid.

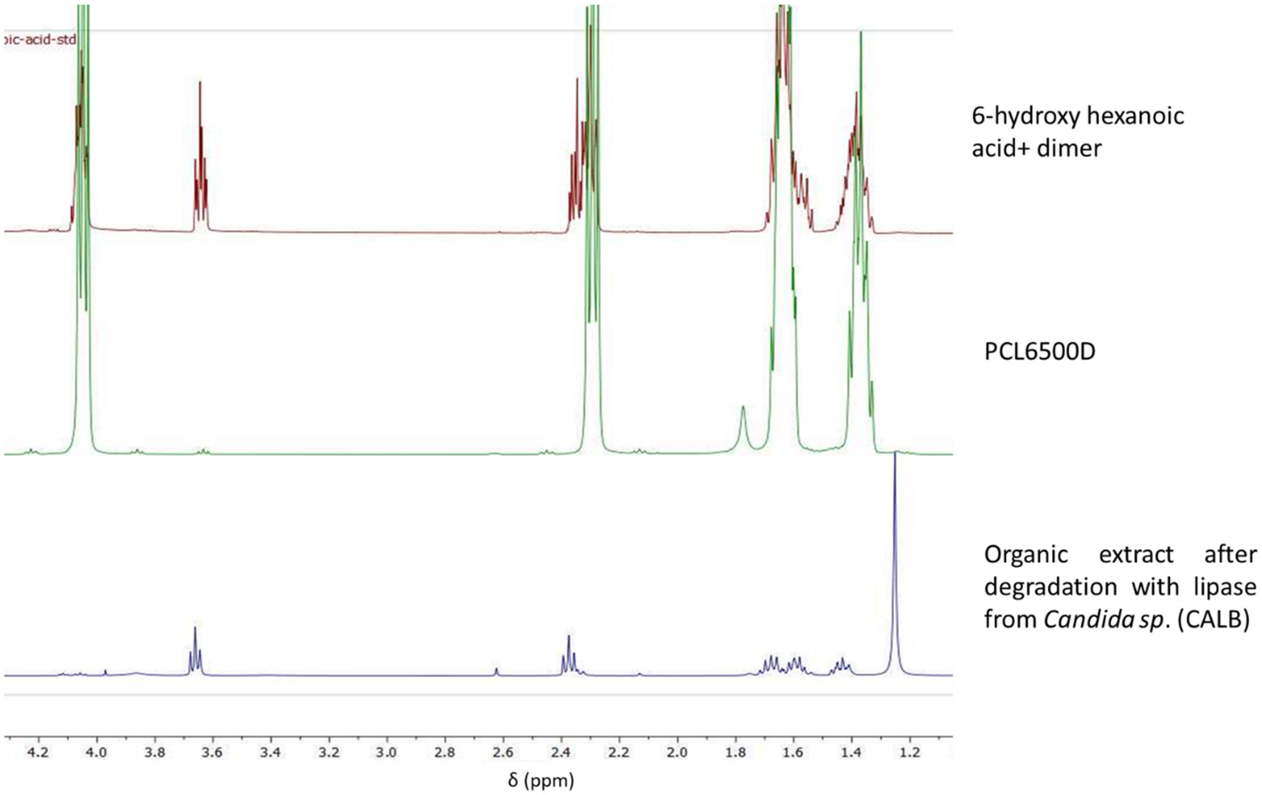

Figure S3d. ^{1}H NMR of the organic extract after degradation of PCL6500D with lipase from *Candida sp*. (CALB), compared to pristine PCL6500D and 6-hydroxyhexanoic acid.

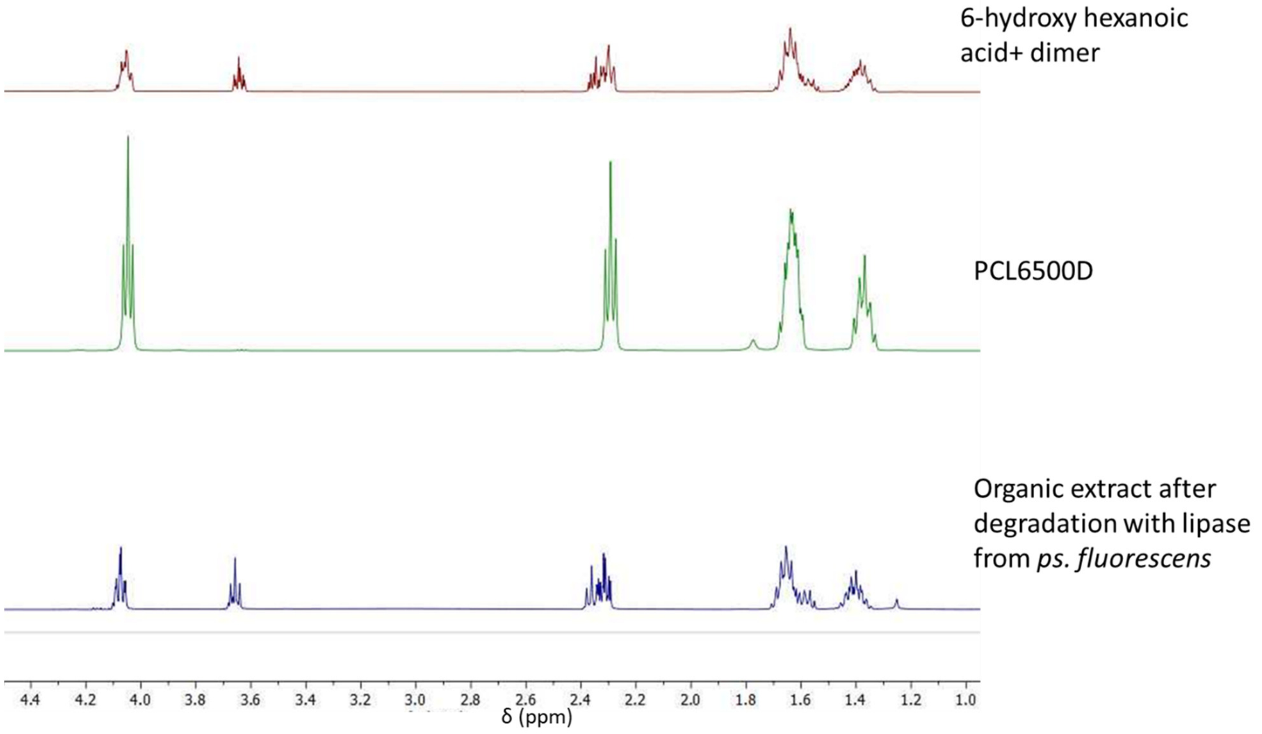

Figure S3e. ^{1}H NMR of the organic extract after degradation of PCL6500D with lipase from *ps. fluorescens*, compared to pristine PCL6500D and 6-hydroxyhexanoic acid.

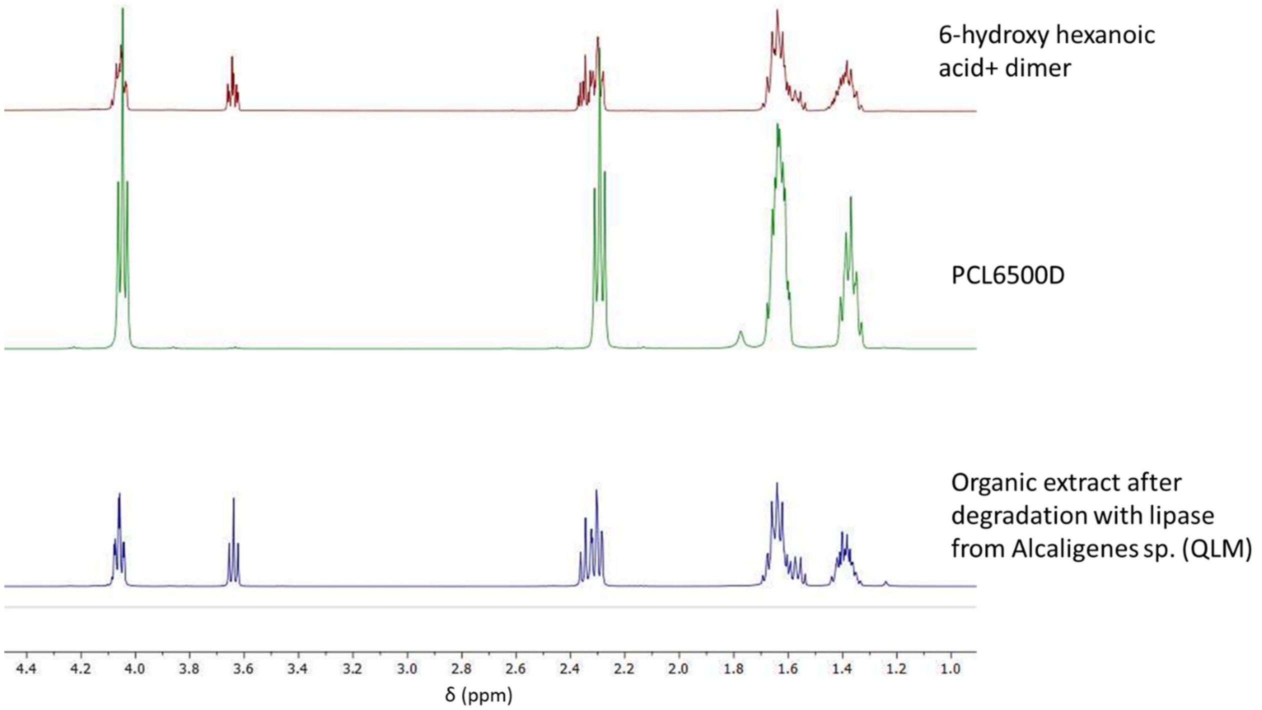

Figure S3f. ^{1}H NMR of the organic extract after degradation of PCL6500D with lipase from *Alcaligenes sp.* (QLM), compared to pristine PCL6500D and 6-hydroxyhexanoic acid.

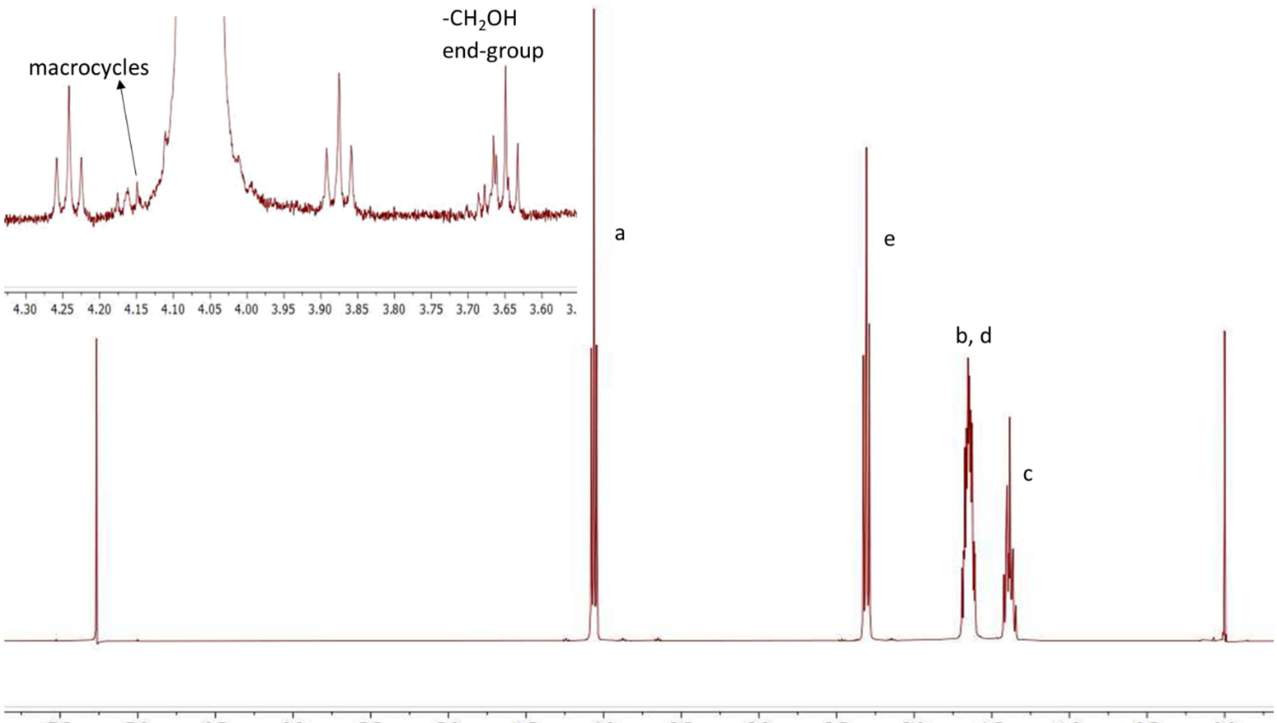

Figure S3g. ^{1}H NMR of PCL6800D incabated with cutinase (4 h).

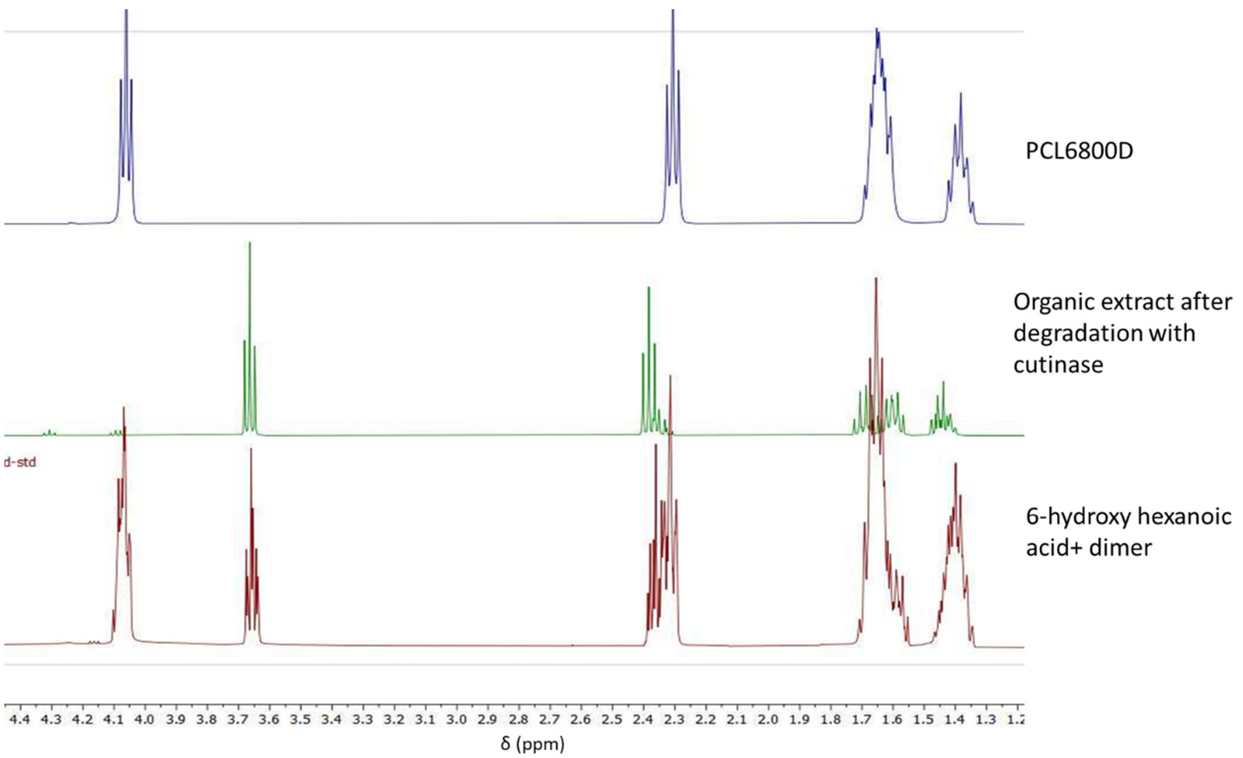

Figure S3h. ^{1}H NMR of the organic extract after degradation of PCL6800D with cutinase, compared to pristine PCL6800D and 6-hydroxyhexanoic acid.

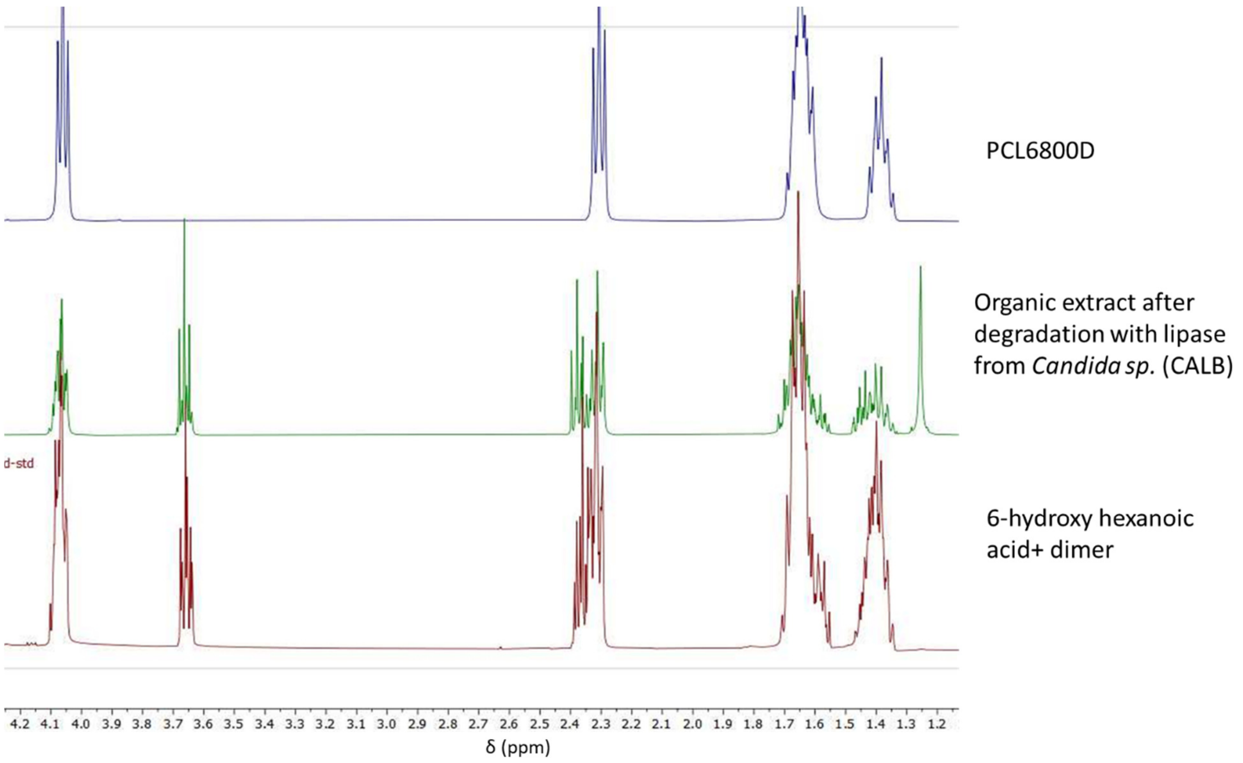

Figure S3i. ^{1}H NMR of the organic extract after degradation of PCL6800D with lipase from *Candida sp.* (CALB), compared to pristine PCL6800D and 6-hydroxyhexanoic acid.

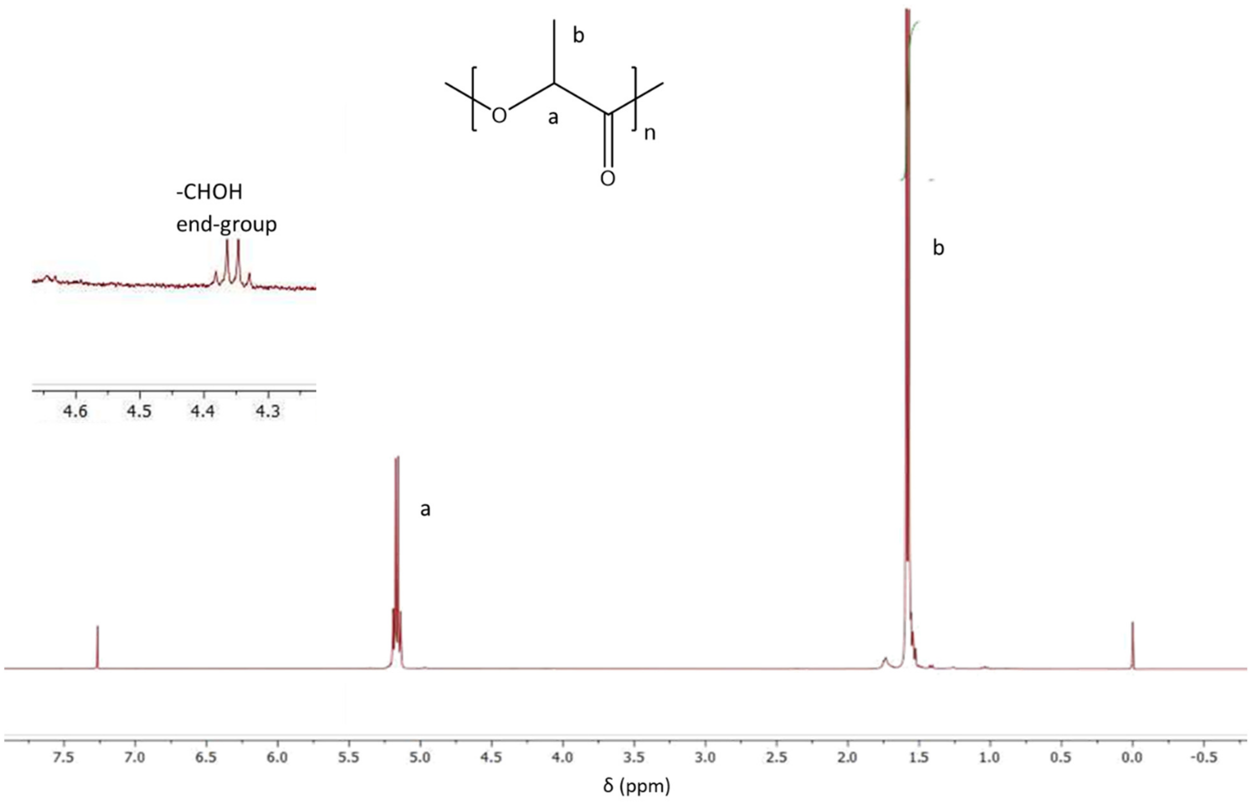

Figure S4. ^{1}H NMR of PLA.

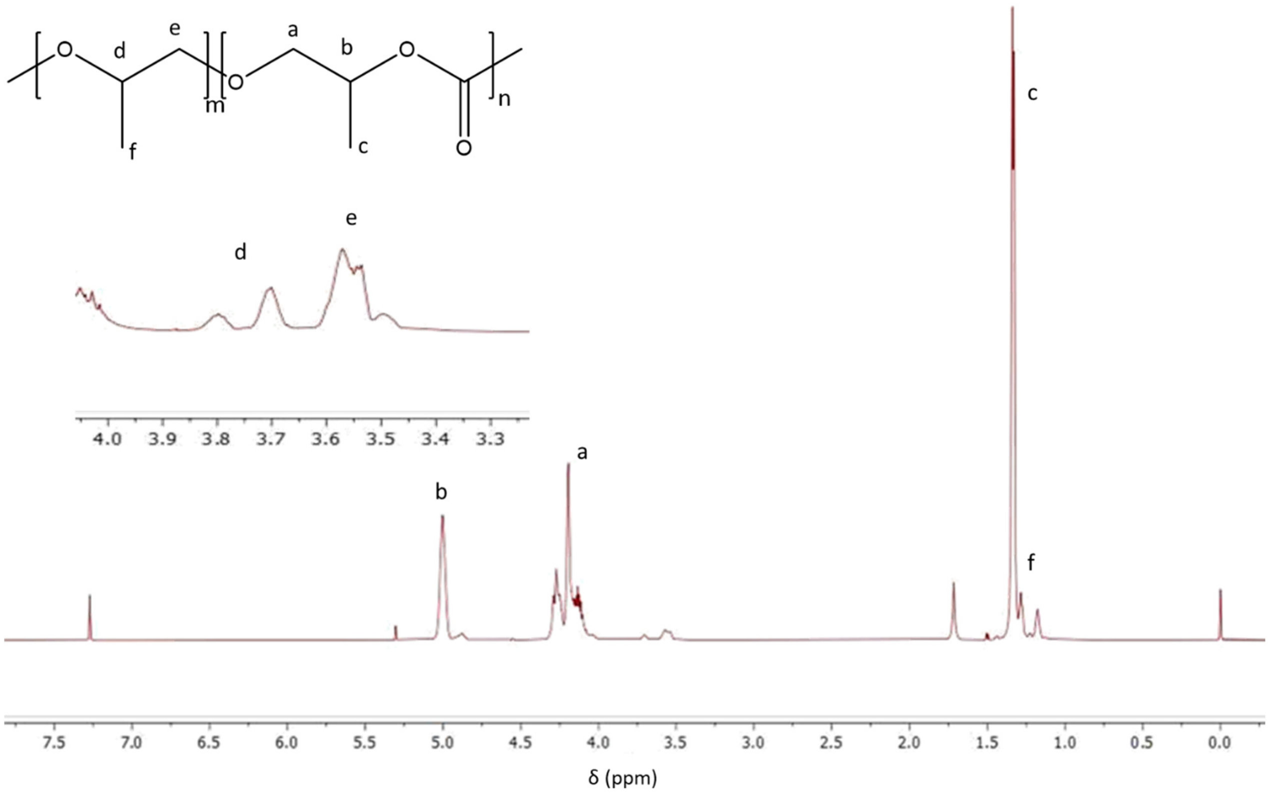

Figure S5a. ^{1}H NMR of PPC.

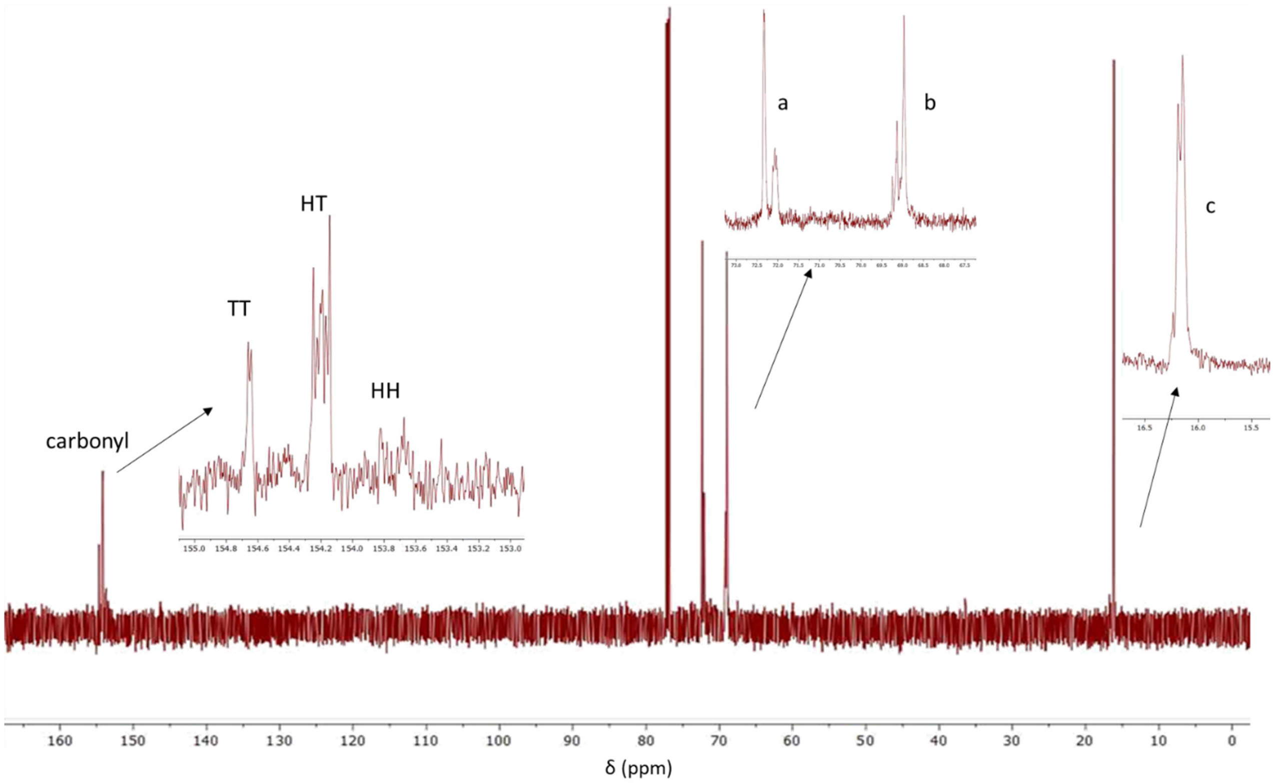

Figure S5b. ^{13}C NMR of PPC.

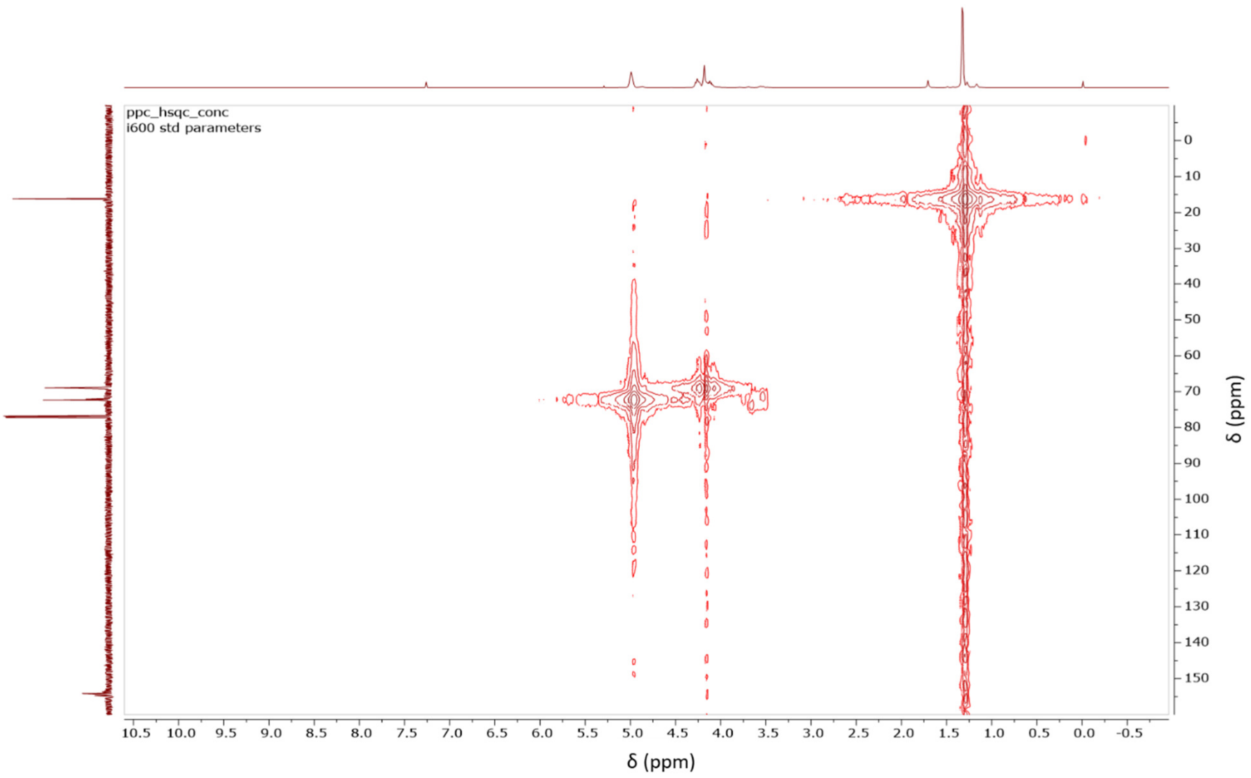

Figure S5c. HSQC (2D NMR) of PPC.

PPC was purified in order to remove propylene carbonate (PC) (although some traces are still detected by ^{1}H-NMR). Considering that the monomer is racemic, the polymer resulted being atactic. Moreover, it is possible to observe on the spectra (both proton and carbon) different regiosequences (Chisholm et al., 2002).

In particular, in the carbonyl region, it is possible to recognize three different series of peaks (see carbon spectrum), corresponding to head-to-head (HH), tail-to-tail (TT) and head-to-tail (HT) regiosequences on a diad level. Moreover, each regiosequence shows more than one resonance that is related to the different regiosequences on a tetrad level (see **Tab. S3**). This is true also for the methine, methylene and methyl carbon regions.

Table S3. Regiosequences of PPC on a diad level and on a tetrad level (Chisholm et al., 2002).

Diad level	Tetrad level
(HT).(HT)	(HT)(HT).(HT)(HT)
	(HT)(HT).(HT)(TH)
	(TH)(HT).(HT)(TH)
	(TH)(HT).(HT)(HT)

- Chisholm, M. H., Navarro-Llobet, D., & Zhou, Z. (2002). Poly (propylene carbonate). 1. More about poly (propylene carbonate) formed from the copolymerization of propylene oxide and carbon dioxide employing a zinc glutarate catalyst. *Macromolecules*, *35*(17), 6494-6504.

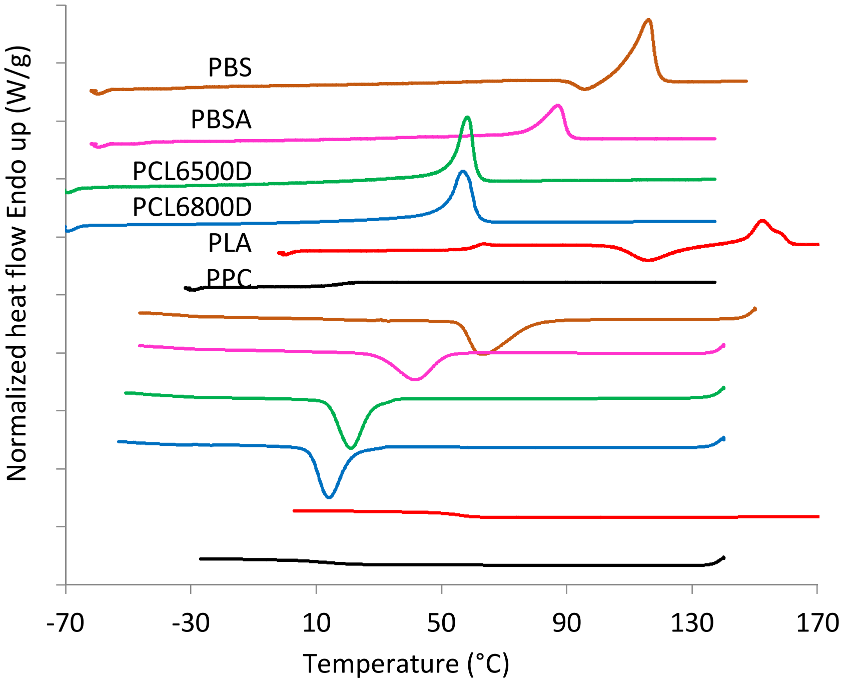

Figure S6. Calorimetric curves of polyesters (second heating scan and cooling from the melt for each type of polyester).

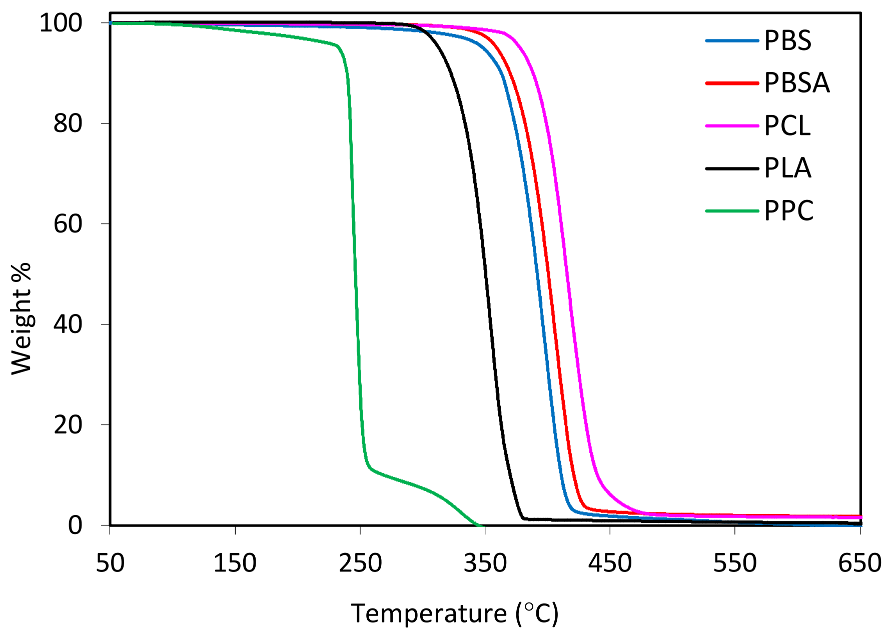

Figure S7a. Thermogravimetric curves of polyesters.

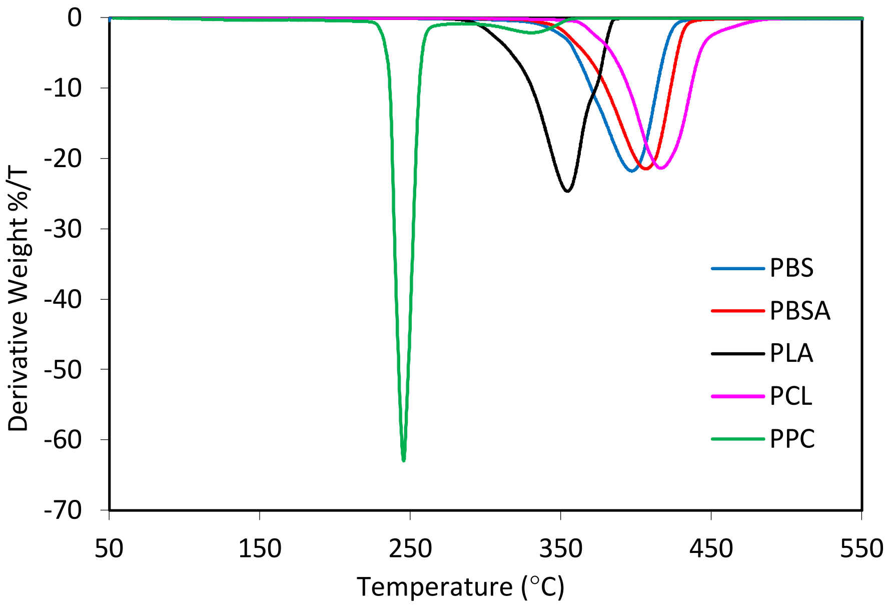

Figure S7b. dTGA curves of polyesters.

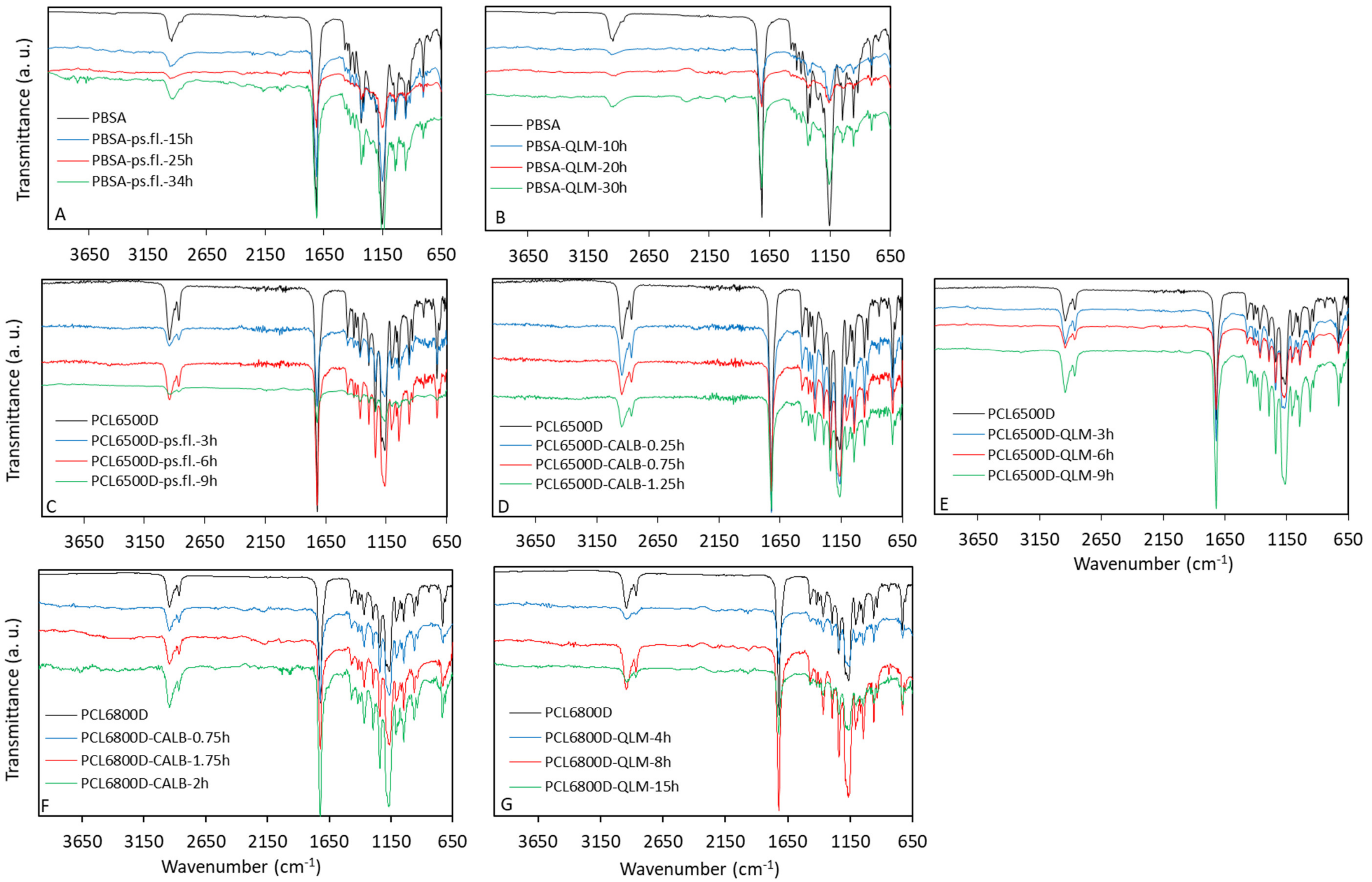

Figure S8. ATR FT-IR curves of polyester residual solids: PBSA after treatment with *Pseudomonas fluorescens* (A) and QLM (B), PCL 6500D after treatment with *Pseudomonas fluorescens* (C), CALB (D) and QLM (E), and PCL 6800D after treatment with CALB (F) and QLM (G).

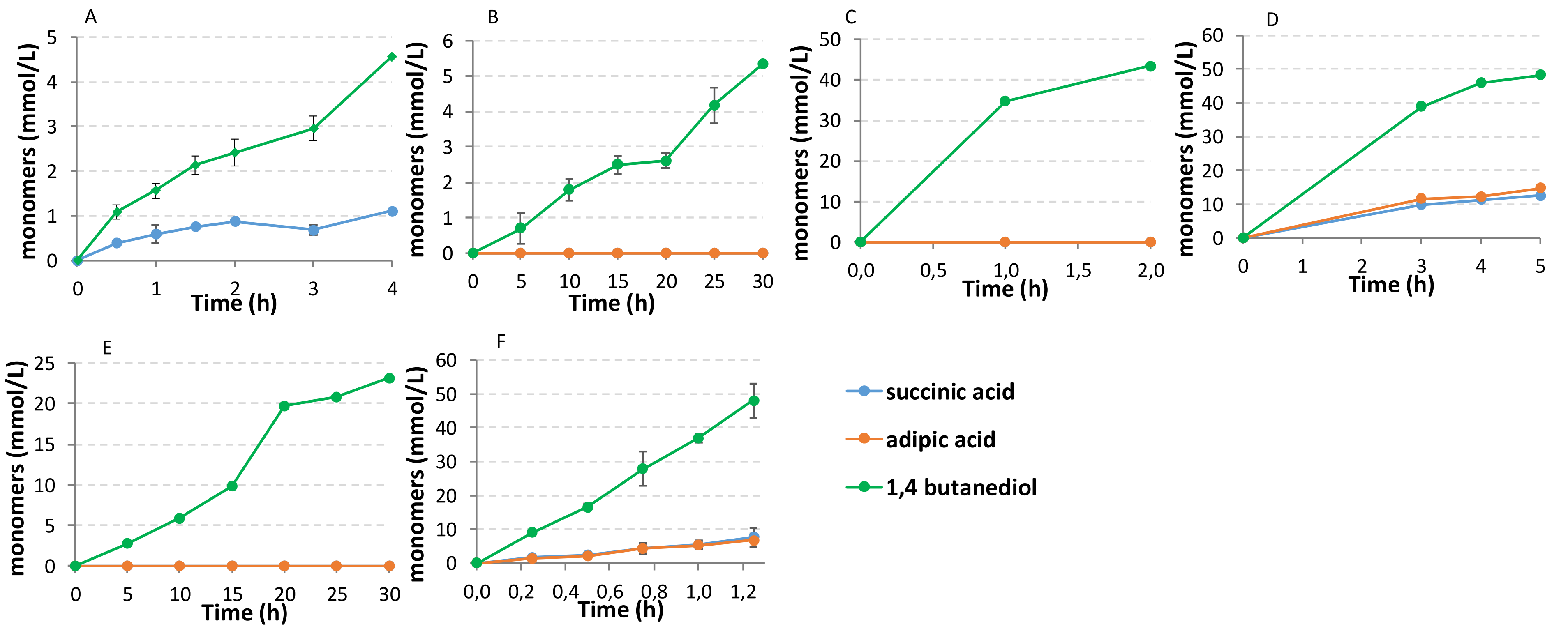

Figure S9. Concentration of succinic acid and 1,4 butanediol (mmol/L) detected via HPLC-RID analysis in the liquid fraction of PBS sample degraded by cutinase (A) over time (hours). Concentration of succinic acid, adipic acid and 1,4 butanediol (mmol/L) detected via HPLC-RID analysis in the liquid fraction of PBSA samples degraded by lipase from *Pseudomonas fluorescence* (B), lipase from *Pseudomonas sp*. (C), lipase B from *Candida antarctica* (D), lipase from *Alcaligenes sp.,* QLM (E) and cutinase (F) over time (hours).
